# FLiP-Z by Zimeck: A Python-Based Machine-Learning Tool for Predicting Fungicide-Likeness of Organic Molecules

**DOI:** 10.3390/ijms27177562

**Published:** 2026-08-24

**Authors:** Cristian A. Cervantes, Ximena Jaramillo-Fierro, José R. Mora

**Affiliations:** 1 Doctorado en Química, Universidad Técnica Particular de Loja, San Cayetano Alto, Loja 1101608, Ecuador; cacervantes1@utpl.edu.ec; 2 Departamento de Research y Divulgación, Zimeck C.L., Calle N74C y Av. Eloy Alfaro, Quito 170307, Ecuador; 3 Departamento de Química, Universidad Técnica Particular de Loja, San Cayetano Alto, Loja 1101608, Ecuador; xvjaramillo@utpl.edu.ec; 4 Department of Chemical Engineering, Universidad San Francisco de Quito, Diego de Robles s/n y Av. Interoceánica, Quito 170157, Ecuador

**Keywords:** QSAR, computational prediction, computational screening, fungicidal activity, QuBiLs descriptors, TOMOCOMD-CARDD, in silico

## Abstract

Development of new agricultural fungicides requires reliable computational tools to screen candidate molecules before investing time, money, and effort in experimental assays. In this study, curated datasets of fungicidal and non-fungicidal compounds were employed in the construction of possible predictive models using diverse machine-learning algorithms and molecular descriptors. The best performance was obtained using balanced datasets and topological descriptors. Two classification models based on different kinds of negative class instances were selected, achieving results of accuracy, sensitivity, and specificity for the test set of 0.806, 0.778, and 0.828 for the first model (RF_BF_14), and 0.937, 0.943, and 0.933 for the second model (RF_BF_17). Differences in performance were consistent with the chemical nature of the negative class. Both models showed excellent applicability domain coverage (>99.6%). Predictions of fungicidal likeness were applied to a curated database of about 1.2 million molecules from the ChEMBL database by using an in-house developed Python3 tool: FLiP-Z (Fungicide Likeness Predictor by Zimeck). Roughly 22% of molecules were predicted by positive consensus as fungicidal candidates. A subsequent screening based on Acute Oral Toxicity reduced the set to 295 molecules with low-toxicity and positive fungicide-likeness predictions. Proprietary rights for FLiP-Z are held by Zimeck C.L.; the tool is available by permission.

## 1. Introduction

Fungal diseases represent one of the most persistent problems in modern agriculture, causing losses in crop yield and quality around the world and generating significant economic impact in the food production chain [1]. The increasing prevalence of phytopathogenic fungi is a serious issue for global food security, particularly in countries experiencing population growth and climate change [2,3]. The continuous emergence of resistant fungal species has complicated this problem, undermining the long-term effectiveness of current disease-control strategies and highlighting the urgent need for novel fungicidal agents [4].

Fungicides have played a central role in the management of fungal diseases in agriculture for a long time [5]. However, the development of new fungicides through traditional experimental approaches is an inherently time- and resource-intensive process, often resulting in compounds that fail to meet necessary physicochemical properties for efficacy and safety [6,7]. In addition to escalating development costs, growing concerns have emerged regarding human toxicity, ecotoxicological effects, environmental persistence, and unwanted impacts on non-target organisms [8,9]. From a mechanistic point of view, effective fungicides are expected to act as protectants, preventives, or curatives, also to target conserved enzymes or proteins across multiple pathogens and display adequate penetration and translocation properties when not operating via contact mode of action [10,11]. Achieving an optimal balance between molecular mobility, stability, and pathogen penetration remains a major challenge in fungicide development for crop protection, often limiting the success of candidate compounds during later development stages [5,6,12].

Experimental screening pipelines frequently fail in advanced phases due to unfavorable physicochemical properties, such as poor solubility and low permeability, leading to late-stage attrition and substantial economic losses [11,13]. These limitations underscore the need for complementary computational strategies to reduce failure rates, prioritizing promising candidates earlier, and rationalizing experimental efforts [14,15,16,17,18].

Quantitative Structure–Activity Relationship (QSAR) modeling, based on machine-learning algorithms, has emerged as a valuable tool for compound prioritization by establishing mathematical relationships between molecular structure and biological activity [15,19,20]. Nevertheless, QSAR approaches are not exempt from limitations. Structural similarity alone does not guarantee similar bioactivity, and model extrapolation beyond the defined applicability domain remains a critical concern [21]. These issues about QSAR in agrochemical fungicide development emphasize the necessity of more integrative and systematic frameworks that combine multiple criteria rather than relying on isolated similarity metrics [14].

In the context of agrochemicals and fungicides, QSAR studies have explored diverse features, datasets, and descriptor families, including agrochemical-specific and pesticide-likeness criteria [7,22,23], classical physicochemical descriptors [15,24,25], hydrophobicity-related indices such as AlogP [26,27], Abraham’s solvation parameters [24], aromaticity-related descriptors [15,28], and fungicide-oriented descriptor sets [7,29,30]. Despite these efforts, methodological heterogeneity, encompassing variability in descriptor selection, dataset curation standards, and validation protocols, and the absence of community consensus have limited the generalization and practical adoption of agro-QSAR models [14,22,23,24,31,32]. For instance, reported fungicide-likeness models have employed datasets ranging from a few hundred to several thousand compounds, with negative classes drawn from structurally heterogeneous libraries, making direct performance comparisons unreliable.

Beyond fungicides, QSAR methodologies have demonstrated notable success in modeling complex biological activities [30,33,34,35]. The QuBiLs methodology, first implemented in the ToMoCoMD-CARDD framework [36] (ToMoCoMD), has been applied to a wide range of bioactivity endpoints, including studies in toxicity [37,38], antimicrobial activity [39,40,41], and other pharmacological targets [42,43]. These applications illustrate the potential of QuBiLs-based descriptors to capture subtle structural features relevant to multifactorial biological phenomena [44].

The present study addresses these limitations through three key contributions. First, we systematically compare two fundamentally different negative class definitions (pharmaceutical drugs versus non-fungicidal agrochemicals) and demonstrate their complementary value in a consensus classification strategy. Second, we apply QuBiLs-MAS topological descriptors, which encode molecular information through algebraic graph-theoretic formalisms, to the fungicide-likeness classification problem for the first time, achieving substantial improvements over classical physicochemical descriptors. Third, we provide FLiP-Z, a Python-based tool capable of large-scale prospective screening, demonstrated here on a library of over 1.2 million molecules. FLiP-Z integrates consensus predictions from two complementary Random Forest models and includes applicability domain compliance, resulting in a more rigorous, multi-tiered screening workflow.

## 2. Results and Discussions

### 2.1. Positive Dataset

The Compendium of Pesticide Common Names (BCPC) has cataloged a total of 486 fungicide names (raw positive class), and during the search of their corresponding SMILES in PubChem (https://pubchem.ncbi.nlm.nih.gov/ accessed on 20 August 2026), some fungicides listed under the “Agrochemical Information” filter, but not included in the BCPC, were added to enhance the dataset. An in-house Python script was then employed to systematically clean the dataset through a pipeline (see Table 1) designed according to the cleaning process described in the Section 3. Around 60 inorganic fungicides were discarded, while others were eliminated because they were identified duplicates after standardizing SMILES and detected mixtures through disconnected fragments. Ultimately, molecules with a molecular weight outside the range of 90 to 700 Daltons, and those with a topological polar surface area (TPSA) of zero were also excluded. The final curated positive class dataset of fungicides consists of 376 instances (see Table S1.1 in Supplementary Information S1).

While having the maximum possible number of positive class entities is beneficial for creating a strong model, it is equally crucial, if not more so, to eliminate any inconsistent or non-applicable molecules, as they could diminish the model’s performance, even if they are few. As shown above, for our purposes, non-applicable fungicides included inorganic salts and similar inorganic-based fungicides, as the aim is to predict the fungicide likeness of molecules with an organic composition.

### 2.2. First Negative Dataset: Drugs from ChEMBL and Internal Validation

Negative class (D): ChEMBL (https://www.ebi.ac.uk/chembl/ accessed on 20 August 2026) is a curated database containing more than 2.5 million compounds associated with one or more bioactivity assays serving as a useful and credible source of promising molecules for drug development research. We initiated the cleaning process with a dataset of 15,591 molecules cataloged by ChEMBL under the term “Drugs”. This number was systematically reduced to approximately one-third by discarding entries with missing SMILES or “USAN definition” data (USAN—United States Adopted Name, unique nonproprietary names assigned to pharmaceuticals marketed in the United States), as well as those with “USAN definition” classified as fungicides, antimicrobials, or antibiotics. Subsequently, the same cleaning pipeline shown in Table 1 and applied to the positive class dataset was executed, removing instances with inorganic compounds, mixtures, non-parsed SMILES, 3D generation failures, and duplicates. The final curated negative class dataset comprised 3728 molecules.

**Figure 1:**
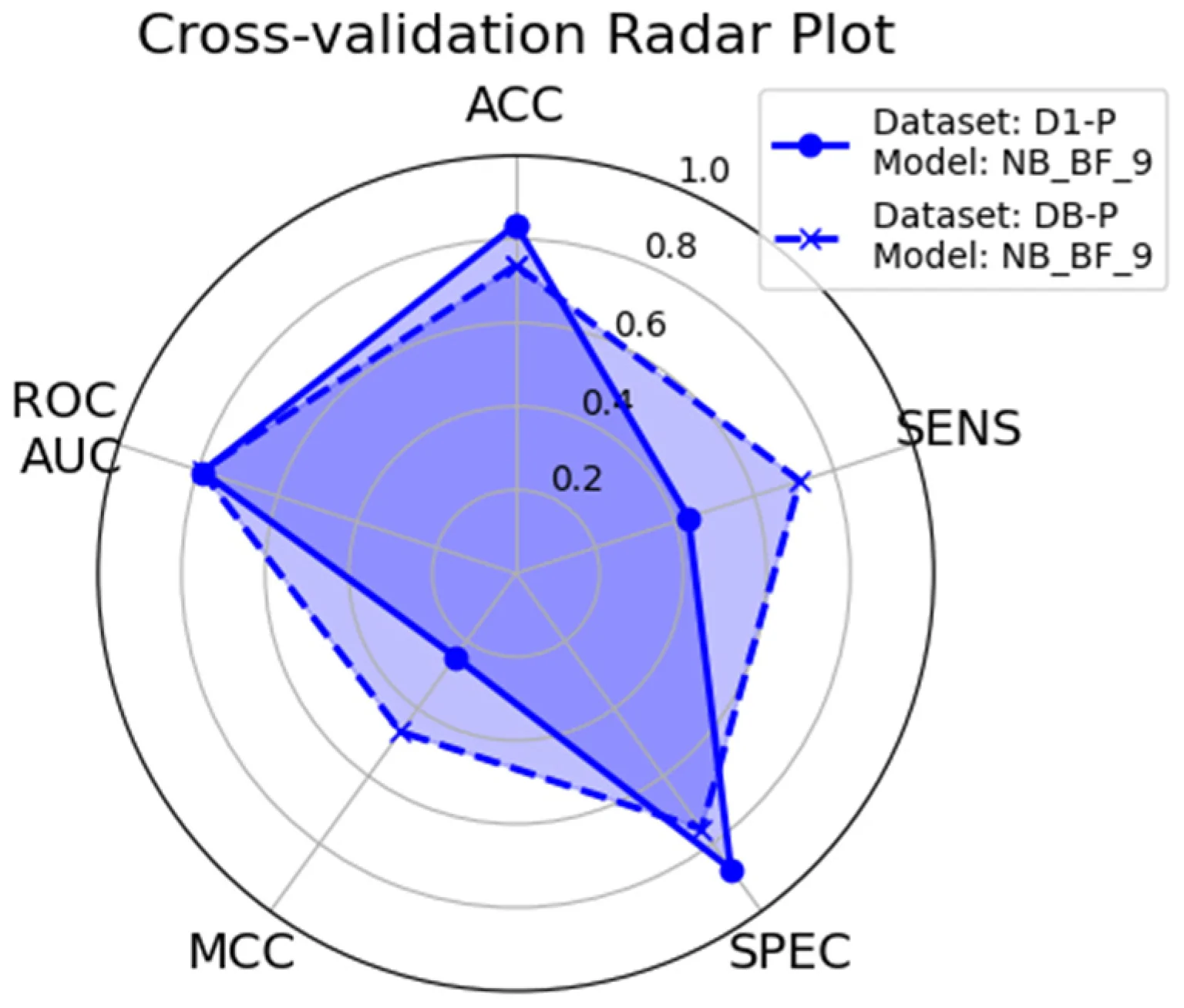
Results of Internal Validation of datasets D1-P (not balanced) and DB-P (balanced). Both results were obtained through a 10-fold cross-validation in Weka, using model NB_BF_9: Naive-Bayes classifier with 9 PhysChem descriptors.

Therefore, our first curated dataset (D1) comprised 4104 molecules (3728 negative and 376 positive instances). This dataset is analogous to that reported by Moriwaki et al. in FungiPAD [7]. However, since the original dataset was not made publicly available by the authors, direct instance-by-instance comparison was not possible, then our initial effort was focused on the construction of a similar model for this dataset. Nine descriptors were selected (PhysChem descriptors. See Section 3) and were calculated using RDKit version 2024.9.6 (https://www.rdkit.org/ accessed on 20 August 2026); dataset was renamed as D1-P, and internal validation was performed employing configurations similar to previous works [7]: through a 10-fold cross-validation using a Naive Bayes classifier implemented in Weka (https://mL.cms.waikato.ac.nz/weka/ accessed on 20 August 2026). This model was called NB_BF_9 (classifier: Naive Bayes; feature selection method: Best First; number of descriptors: 9; molecule-to-attribute ratio: approximately 450:1). Performance of the model was evaluated through six parameters: accuracy (ACC), sensitivity (SENS), specificity (SPEC), Matthews’ correlation coefficient (MCC), and area under the receiver operating characteristic curve (ROC-AUC), as explained in the Section 3. The obtained ACC of 0.835 and high ROC-AUC value were consistent with expectations. However, the SENS of 0.434 and SPEC of 0.875 revealed a model bias toward predicting negative instances, which is problematic for the identification of new lead molecules, as potentially viable candidates may be discarded as false negatives. Other metrics, such as the MCC, also indicated this limitation (see Figure 1), while other results showed that alternative classifiers could have been selected instead of Naive Bayes (see Supplementary Information S2).

A second internal validation was performed using a sampled dataset that improves the ratio between positive and negative instances (sampling criteria are detailed in Section 3). An almost balanced dataset (DB-P) was constructed comprising 499 sampled negative instances and all 376 positive instances achieving a ratio of 1.33:1 (for simplicity, the term “balanced” will be used despite ratio is not exactly 1:1). The results showed overall improvement (see Figure 1), with SENS increasing to 0.715 and MCC achieving considerably better values, although ACC (0.738) and SPEC (0.756) decreased moderately compared to the imbalanced dataset D1-P. Notably, the ROC-AUC, which has been used as the primary decision criterion [7], remained nearly unchanged despite the balancing approach.

**Figure 2:**
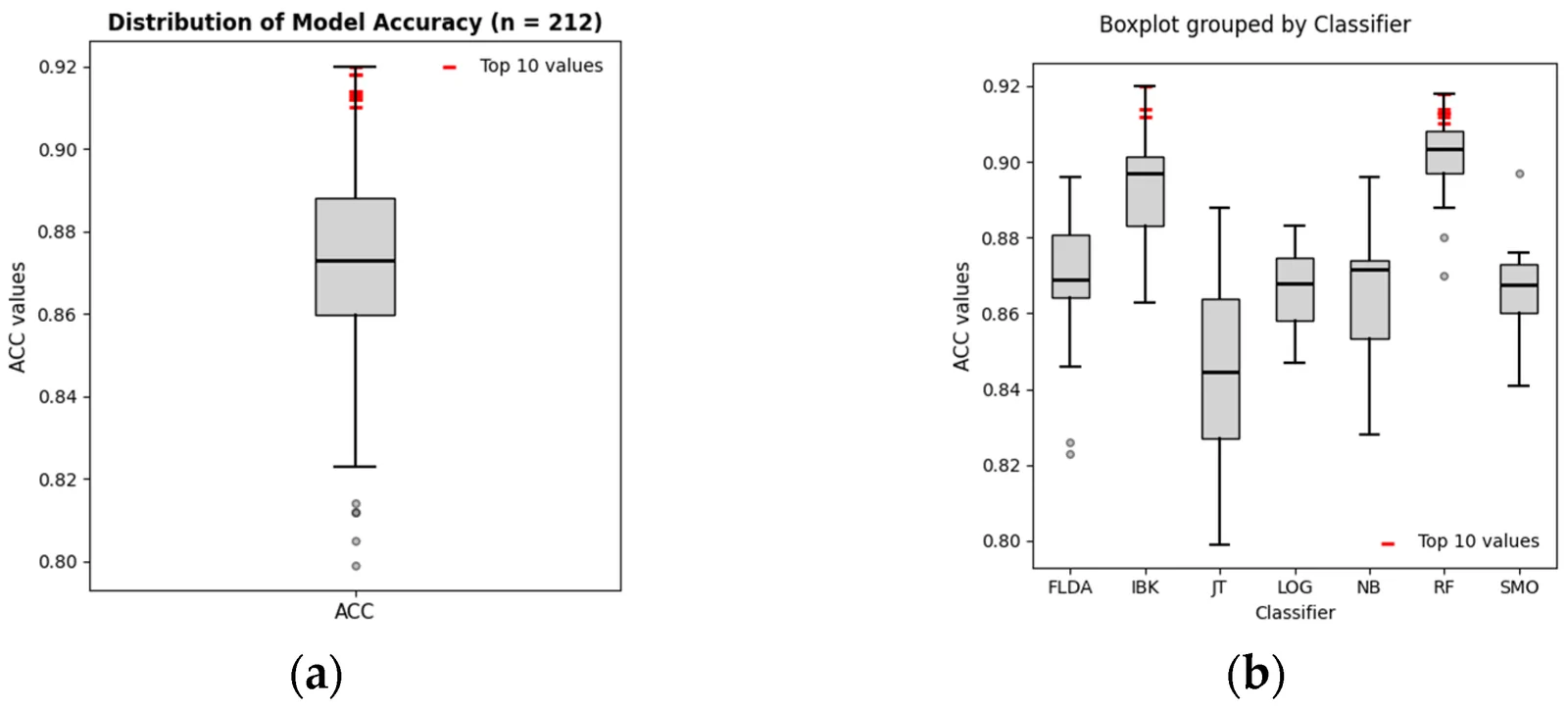
Top 10 Models for dataset DB-Q, Ranked by Accuracy (ACC). Each boxplot represents the distribution of accuracies, with red lines indicating the positions of the top 10 models within that distribution. (**a**) All evaluated models; (**b**) evaluated models grouped by classifier.

**Figure 3:**
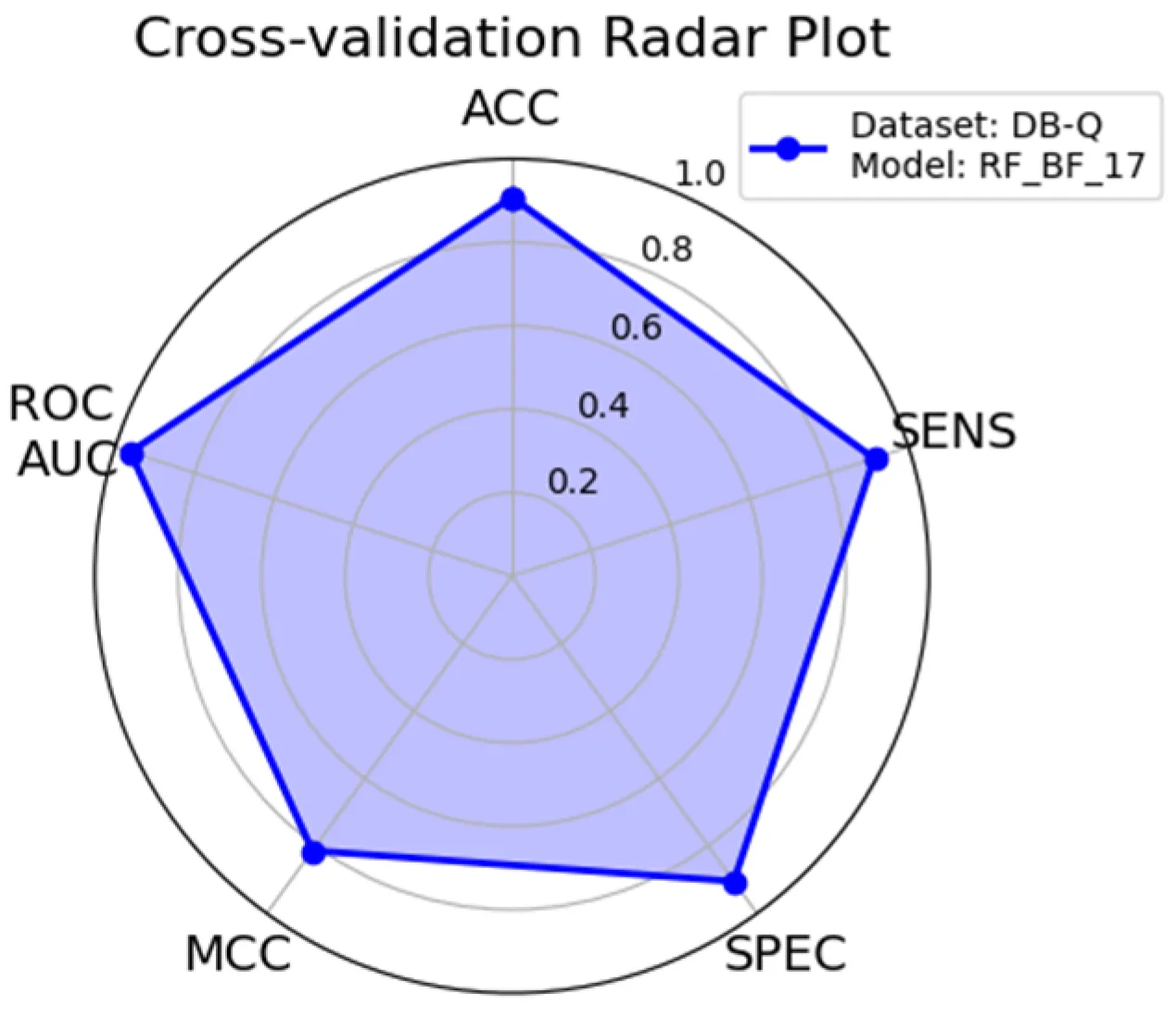
Results of Internal Validation of dataset DB-Q. Results were obtained through a 10-fold cross-validation in Weka, using model RF_BF_17: Random Forest classifier with 17 QuBiLs-MAS descriptors. ACC = 0.908, SENS = 0.913, SPEC = 0.905, MCC = 0.814, ROC-AUC = 0.962.

The balanced dataset was then used to evaluate models constructed with QuBiLs-MAS descriptors, which have demonstrated good results in other applications [37,38,39,40,41,42,43,44]. After obtaining the 3D coordinates of each molecule in the dataset, 836 QuBiLs-MAS descriptors were calculated using the software QuBiLs-MAS v1.0 (https://tomocomd.com/software/qubils-mas accessed on 20 August 2026) from ToMoCoMD framework. This number was reduced to 423 descriptors through a collinearity analysis based on Pearson correlation coefficients (threshold: 0.7) using scikit-learn (https://scikit-learn.org/ accessed on 20 August 2026). These descriptors were used in Weka to construct 212 models that were combined with various meta-classifiers (see Section 3); these results are available in Supplementary Information S3. The 10 best models ranked by accuracy were identified (see Figure 2), and a new cross-validation step was performed to select the optimal model. The best-performing model was RF_BF_17 (classifier: Random Forest; feature selection method: Best First; number of descriptors: 17; molecule-to-attribute ratio: approximately 51:1), which exhibited very good performance across all metrics (see Figure 3 and Supplementary Information S4). This model result presents a better performance than that for model NB_BF_9, which was obtained with the physicochemical parameters. The balanced dataset filled with these QuBiLs descriptors was named DB-Q.

### 2.3. Second Negative Dataset: Agrochemicals from PubChem and Internal Validation

Negative class (A): A second approach was undertaken to broaden the scope of the evaluated models, based on the possibility of having a dataset with molecule structures analogous to the positive class and, therefore, being able to separate the classes even though the molecular similarity, which can be a stricter way of identifying new possible fungicides. A dataset was retrieved from PubChem and subsequently processed following a procedure analogous to that applied to the ChEMBL database. Beginning with 3022 molecules listed in the Table of Contents for the “Agrochemical Information” collection, the 486 fungicides originally used to construct the positive dataset were carefully identified and excluded. In addition, all entries explicitly tagged as “fungic” were removed. Further data cleaning involved eliminating SMILES strings lacking carbon atoms, duplicate records, and non-numerical CID codes (e.g., “NaN”, “?”, and similar anomalies). After these refinements, the resulting negative dataset comprised 2681 instances.

**Figure 4:**
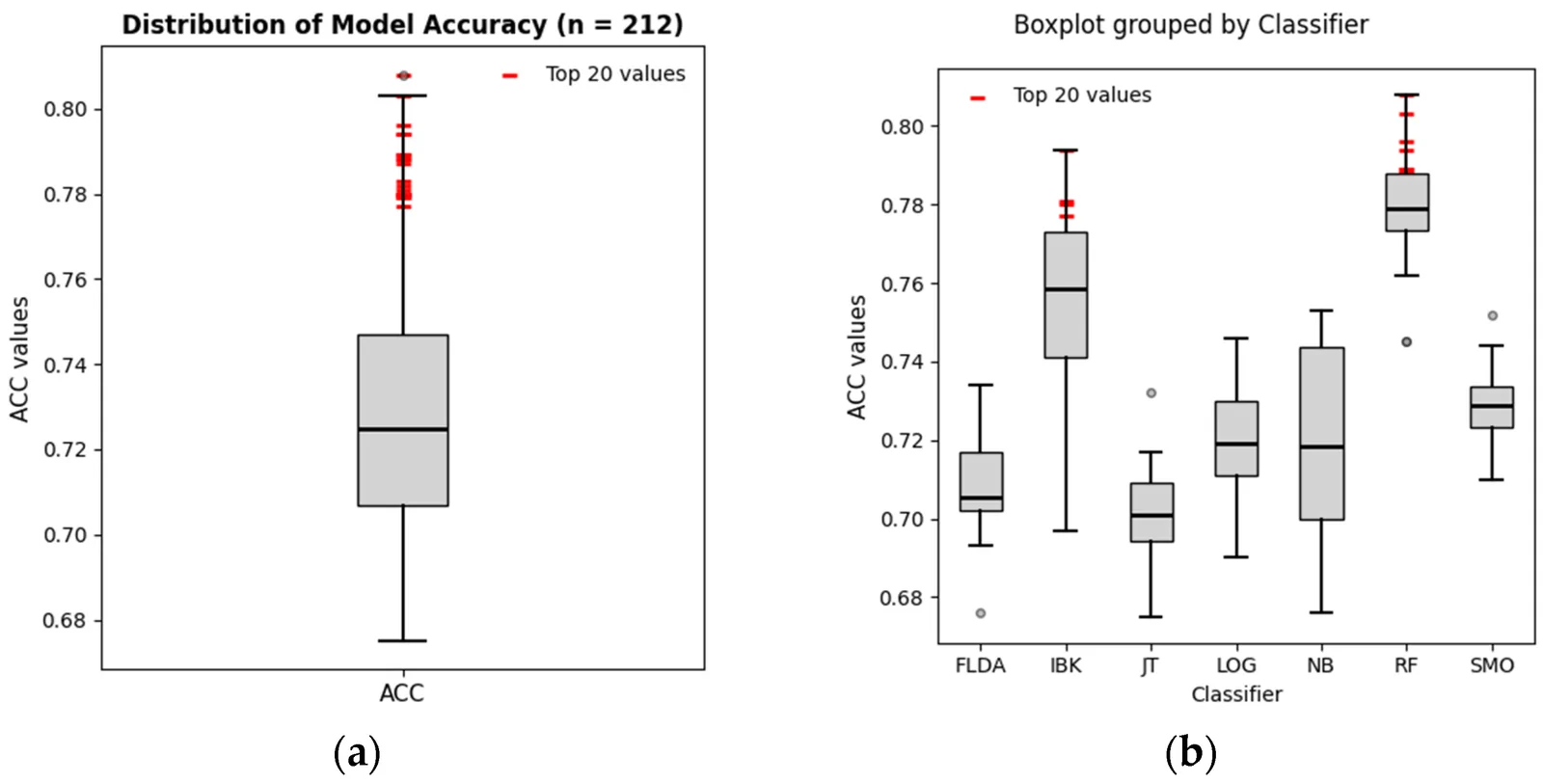
Top 20 Models for dataset AB-Q, Ranked by Accuracy (ACC). Each boxplot represents the distribution of accuracies, with red lines indicating the positions of the top 20 models within that distribution. (**a**) All evaluated models; (**b**) evaluated models grouped by classifier.

**Figure 5:**
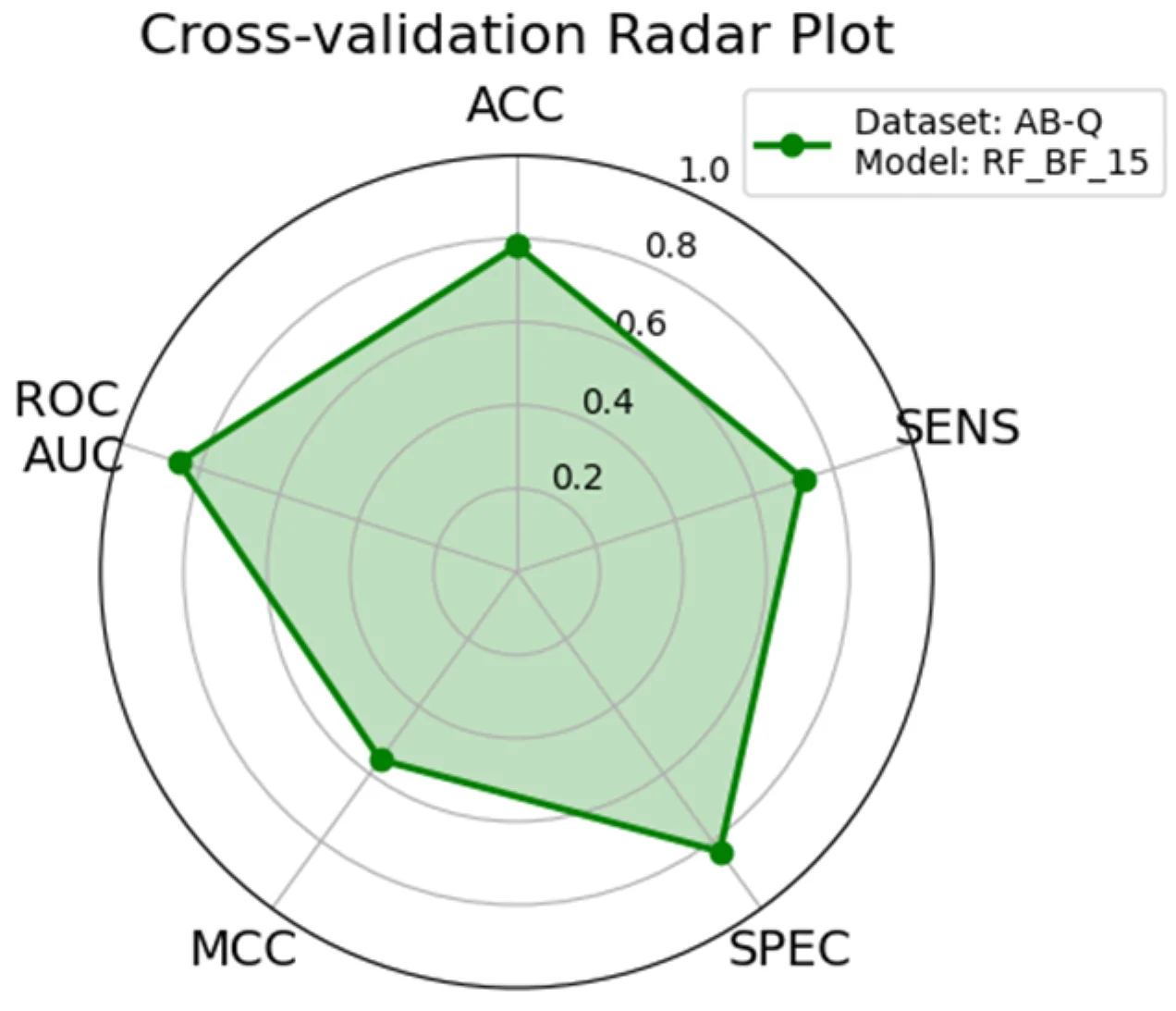
Results of Internal Validation of dataset AB-Q. Results were obtained through a 10-fold cross-validation in Weka, using model RF_BF_15: Random Forest classifier with 15 QuBiLs-MAS descriptors. ACC = 0.783, SENS = 0.723, SPEC = 0.831, MCC = 0.558, ROC-AUC = 0.853.

To obtain the second curated dataset (A1), positive and negative instances were merged, and balanced datasets (AB-P, AB-Q), both with 482 negative and 376 positive instances, were generated using a sampling procedure analogous to that applied previously. As before, the calculated descriptor types were the same nine PhysChem descriptors leading to the established model NB_BF_9, and approximately 400 QuBiLs-MAS descriptors derived from the new set of molecular coordinates. For the latter, model construction in Weka followed identical criteria, yielding 212 models combined with the same meta-classifiers (see Supplementary Information S3). In this case, 20 models were evaluated (twice as many as in the earlier dataset) because accuracy values were comparatively lower but acceptable (see Supplementary Information S4). The best-performing models were subsequently identified by ranking according to accuracy (Figure 4). Following cross-validation, the optimal model for the agrochemical collection was selected. The best-performing model, RF_BF_15 (classifier: Random Forest; feature selection method: Best First; number of descriptors: 15; molecule-to-attribute ratio: approximately 57:1), demonstrated solid performance across all metrics (Figure 5), although consistently lower than the top-performing model obtained from the drugs-based dataset DB-Q. Notably, the agrochemical-based model ranked lower in cross-validation results, reflecting a stricter separation of classes; this outcome is attributable to the greater structural similarity between negative-class agrochemical molecules and the positive-class fungicides.

### 2.4. External Validation with Selected Models

As outlined in the Section 3, the training of the selected models was conducted using multiple datasets that differed in the origin of the negative-class molecules, in the balance of positive and negative instances, and in the type of descriptors employed. In total, 12 working datasets were generated and subsequently partitioned into training and test sets with a 70:30 ratio using procedures designed to preserve the statistical properties of the curated dataset in both subsets. The 12 test sets were reserved for later validation, while the 12 training sets were used to develop the respective trained models based in Random Forest training approach when datasets were processed with QuBiLs-MAS descriptors, or based on the Naive Bayes approach when PhysChem descriptors were included for comparative analysis (see Table 2).

Once the respective models were successfully trained, external validation was performed using the 12 reserved test datasets. Each test dataset was evaluated in Weka against its corresponding trained model. A figure with the detailed results of this stage is provided in Supplementary Information S1 (Figure S1.1).

The first outcome of the external validation revealed that the models RF_BF_17 and RF_BF_15 outperformed the model NB_BF_9. The Naive Bayes (NB) classifier, which utilizes a probabilistic learning approach based on Bayes’ theorem [45], was applied to datasets using PhysChem descriptors with the objective of replicating models previously established for assessing fungicide-likeness in recent works [7]. However, as indicated in the Supplementary Information S2, other models utilizing different machine-learning approaches could enhance the performance of PhysChem descriptors. Conversely, for datasets employing QuBiLs-MAS descriptors, the Random Forest (RF) algorithm, which constructs an ensemble of decision trees and aggregates their predictions [46], was selected through a modeling process aimed at identifying the most effective machine-learning approach for these descriptors. These findings underscore the importance of selecting appropriate algorithms for different descriptor types, as demonstrated by the superior performance of RF over NB in this context.

The second-most notable outcome at this stage was that all balanced datasets consistently outperformed the unbalanced ones (see Supplementary Information S5). This finding guided the decision to prioritize balanced datasets in the subsequent optimization steps.

### 2.5. Quality and Importance of the Descriptors

**Figure 6:**
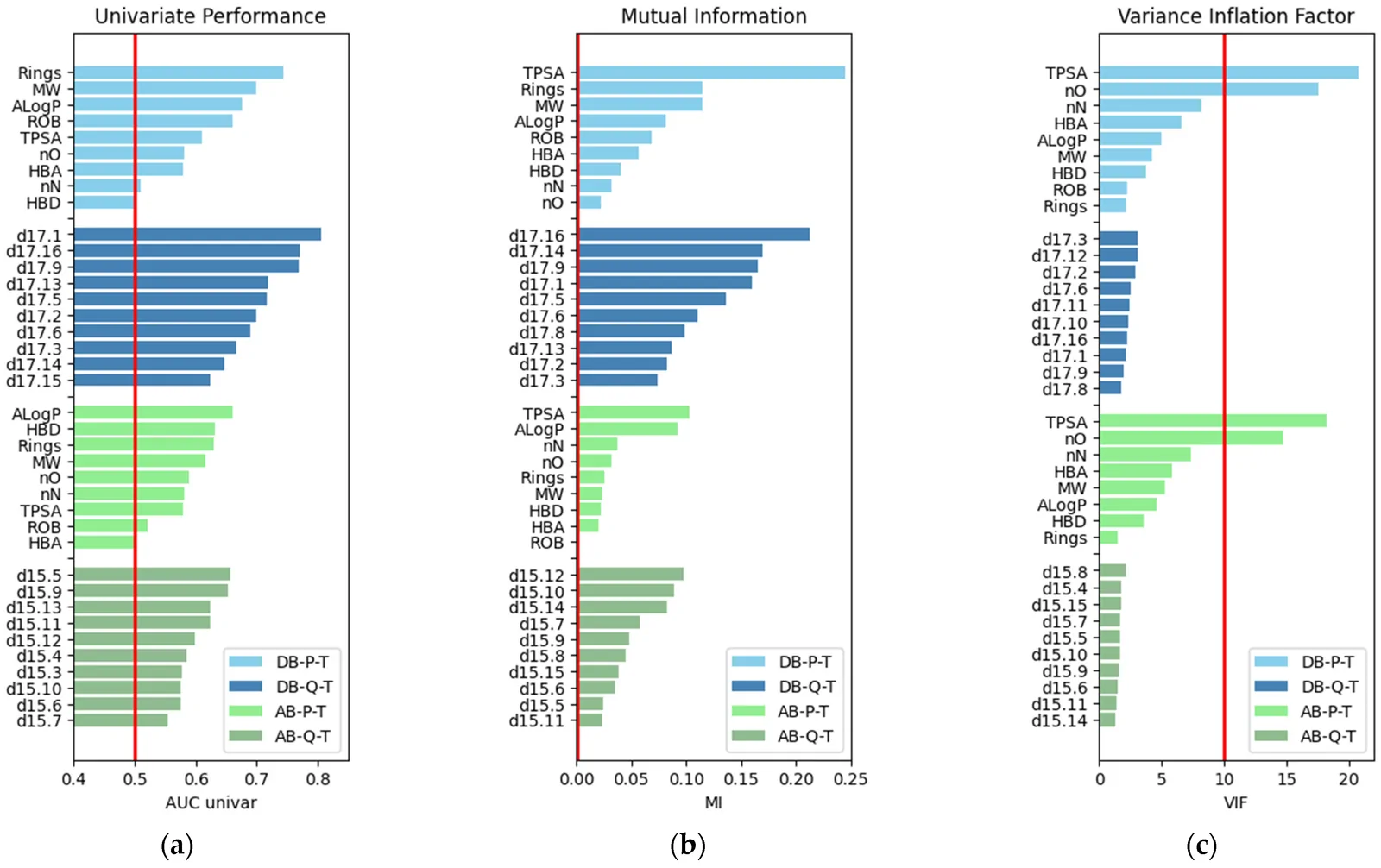
Multivariate analysis of descriptors on datasets DB-P-T, DB-Q-T, AB-P-T, and AB-Q-T. (**a**) Univariate performance (AUC univar); (**b**) Mutual information (MI); (**c**) Variance inflation factor (VIF). Drug-based datasets are shown in shades of blue, and agrochemical-based ones are shown in shades of green. Vertical axis shows the corresponding descriptors in each model; a maximum of 10 descriptors is shown in descending order of their values. Red lines represent the corresponding thresholds that each parameter must meet; descriptors with AUC univar < 0.5, MI = 0, or VIF > 10 should be discarded. Full names of d17 and d15 descriptors are listed in Supplementary Information S1 (Tables S1.2 and S1.3).

For the models associated with the four balanced datasets (DB-P-T, DB-Q-T, AB-P-T, AB-Q-T), a graphical assessment of descriptor quality, based on distribution curve analysis, is provided in the Supplementary Information S1 (Figures S1.2–S1.5). This analysis revealed that PhysChem descriptors include certain discretized populations, such as HBD, nN, and Rings, whereas QuBiLs-MAS descriptors exhibit continuous distributions, consistent with their algebraic nature. In addition, both descriptor types contain populations skewed toward one side of the distribution. To evaluate their relevance to model performance, a multivariate analysis was conducted using the following parameters: univariate performance (AUC univar), which measures the individual predictive power of each descriptor; mutual information (MI), which identifies redundancy between descriptors; and variance inflation factor (VIF), which quantifies the degree of correlation among descriptors [47,48]. The results of this analysis are presented in Figure 6.

Models based on PhysChem descriptors exhibited more quality-related issues. In dataset DB-P-T, seven of nine descriptors were retained, while TPSA and nO were discarded due to variance inflation factor (VIF) values greater than 10, indicating high correlation with other descriptors. In dataset AB-P-T, six of nine descriptors were recommended; the discarded descriptors were ROB, owing to both non-discriminant power (AUC univar ≈ 0.5) and low relevance (MI ≈ 0), as well as TPSA and nO (VIF > 10). For models based on QuBiLs-MAS descriptors, only in the agrochemical dataset AB-Q-T, one descriptor (d15.3) was excluded due to low relevance (MI ≈ 0). In the dataset DB-Q-T, all 17 descriptors were retained. After eliminating the non-recommended descriptors, new models were designated NB_BF_7, NB_BF_6, RF_BF_14, and RF_BF_17, respectively.

As models RF_BF_14 and RF_BF_17 contain the proposed QuBiLs-MAS descriptors to perform the fungicide-likeness predictions (Naive Bayes-based models are studied for comparative reasons), they were evaluated through a y-randomization robustness test applied to the corresponding training datasets (DB-Q-T, AB-Q-T) and obtained the following nested cross-validation results:

- Model RF_BF_14. The original performance for this model was substantially higher than the results obtained from 100 y-randomized iterations: ACC: Original 0.762 vs. Randomized 0.498 ± 0.026; ROC-AUC: Original 0.851 vs. Randomized 0.497 ± 0.035; MCC: Original 0.529 vs. Randomized −0.004 ± 0.056; *p*-value: The empirical permutation *p*-value for all metrics was *p* < 0.01.
- Model RF_BF_17. This model showed even stronger predictive power while maintaining a clear separation from the chance-level randomized models: ACC: Original 0.898 vs. Randomized 0.500 ± 0.024; ROC-AUC: Original 0.952 vs. Randomized 0.500 ± 0.032; MCC: Original 0.793 vs. Randomized −0.001 ± 0.053; *p*-value: The empirical permutation *p*-value was also *p* < 0.01.

The fact that randomized models yield performance close to chance level while the original models remain substantially higher confirms that the models are capturing genuine descriptor–activity relationships. This strongly suggests that the predictive performance is not due to chance correlations, descriptor artifacts, or class imbalance.

**Figure 7:**
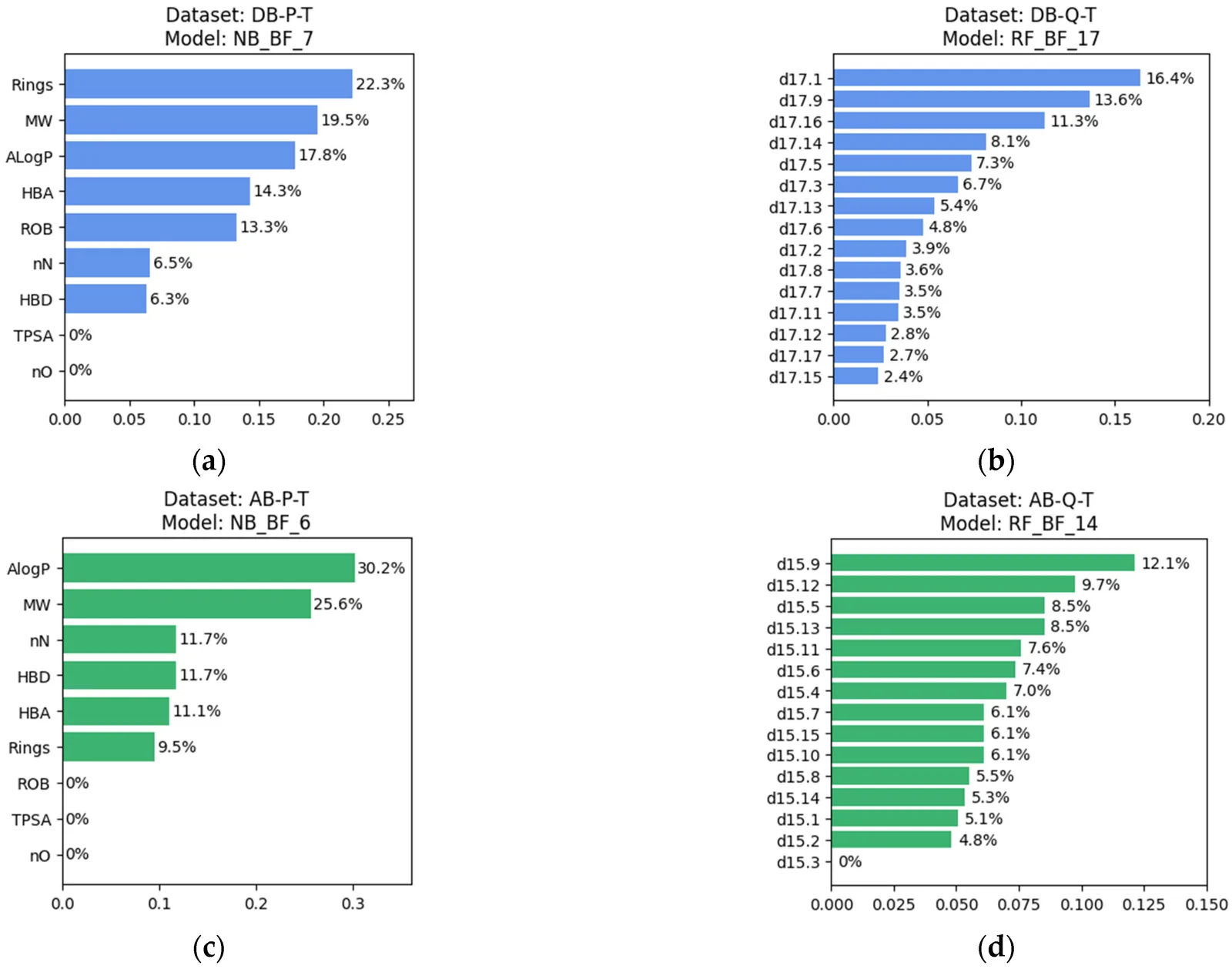
Percentage of the importance of individual descriptors in each optimized model. (**a**) Dataset DB-P-T; (**b**) Dataset DB-Q-T; (**c**) Dataset AB-P-T; (**d**) Dataset AB-Q-T. Discarded descriptors are shown with 0% of importance. Drug-based datasets are shown in blue and agrochemical-based ones are shown in green. Full names of d17 and d15 descriptors are listed in Supplementary Information S1 (Tables S1.2 and S1.3).

As an additional outcome of this analysis, the importance levels of the individual descriptors in each model were also obtained (see Figure 7), allowing a more detailed appreciation of their relevance within the models.

Regarding the descriptors presented in the models RF_BF_14 and RF_BF_17, it is important to point out that QuBiLs-MAS descriptors are not only of algebraic nature but also include some physicochemical features. These topological descriptors are weighted by the following physicochemical properties: electronegativity (e), charge (c), hardness (h), polarizability (p), topological polar surface area (psa), refractivity index (r), Van der Waals volume (v), mass (m), softness (s), and AlogP (a) [36]. In this context, the highest-importance features, accounting for around 40% of total importance in their respective models, are d17.1: a-e, d17.9: p-h, d17.16: c (see Figure 7b and Table S1.2); and d15.9: psa-h, d15.12: c, d15.5: h, d15.13: a-c (see Figure 7d, and Table S1.3). These descriptors encode the physicochemical properties AlogP, electronegativity, polarizability, hardness, charge, and topological polar surface area, features that are consistent with the known requirement for fungicides to interact with conserved enzyme active sites across multiple pathogens [10].

The properties charge, polarizability, and electronegativity suggest the presence of functional polar groups (could include amines, alcohols, amides, and/or carboxylic acids), which agree with the structures presented in fungicide databases; these functional polar groups are related to the formation of charge-transfer complexes in the biological activity of these compounds [49,50]. Hardness and softness are associated with the interactions and energy gaps between the highest occupied molecular orbital (HOMO) and the lowest unoccupied molecular orbital (LUMO) [51,52]. Van der Waals volume and the mass play a pivotal role in reliable coupling with the active site [53,54].

### 2.6. Optimization of Models: Applicability Domain

**Figure 8:**
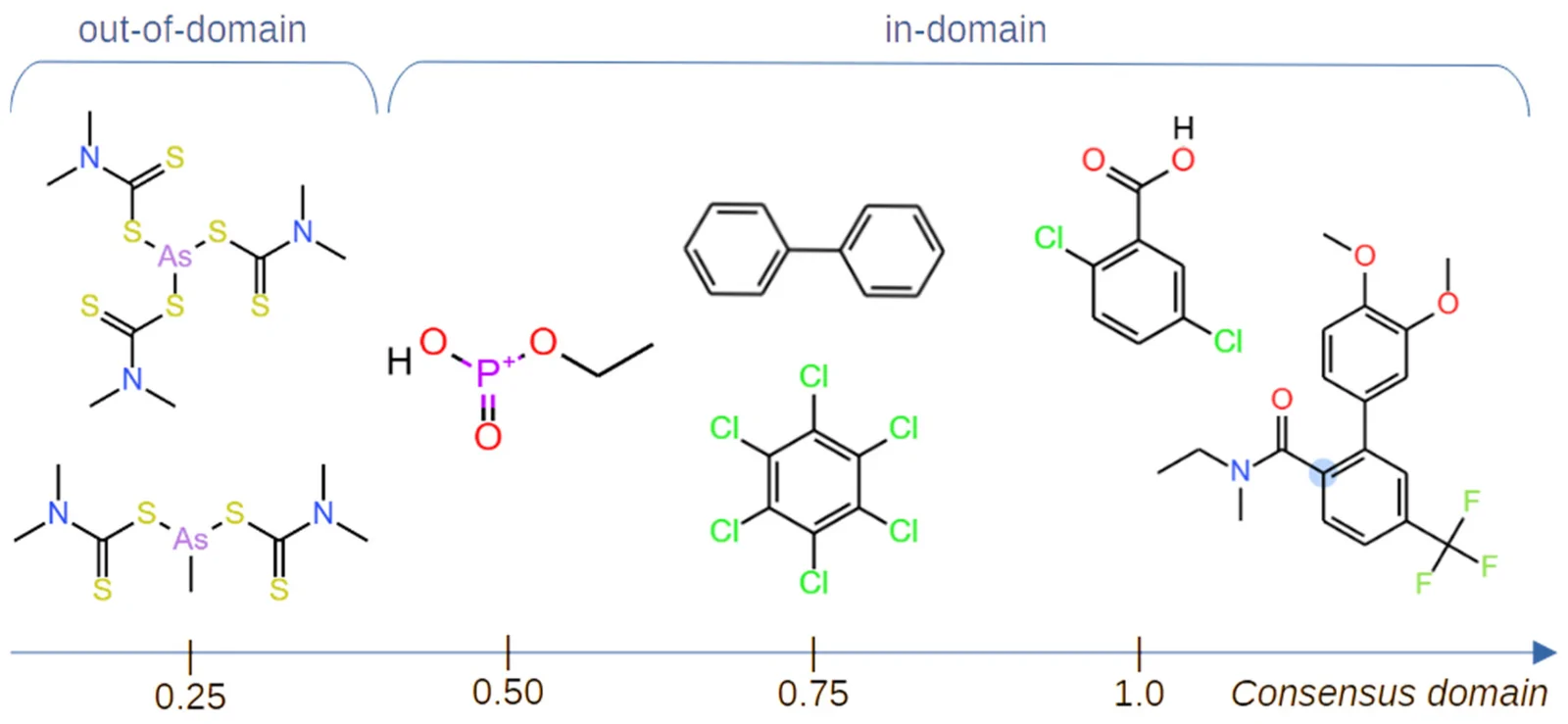
Chemical structure of some in-domain and out-of-domain fungicides. Fungicides in the positive class dataset are shown in order of their “consensus domain”, a metric that quantifies the acceptance level into the four applied methods. A consensus domain equal or less than 0.25 accomplish the consensus rule: more than two methods with out-of-domain results (1 in-domain/4 methods).

The second stage of the optimization process focused on the evaluation of the Applicability Domain (AD). The importance of AD has been established by various authors [55,56,57,58]. In this step, the four balanced training datasets were used as reference sets to assess the AD of the 12 test datasets. The software Ambit Discovery v0.0.4 (https://ambit.sourceforge.net/) was employed for this task, applying the following methods: Ranges (in-domain: normalized violation index < 1.0), Euclidean distance to the mean (in-domain: *d_Euc_* ≤ *D_c_*, where *D_c_* is the maximum distance enclosing the training set), City-block distance to the mean (in-domain: *d_CB_* ≤ *D_c_*), and Probability density (in-domain: *P(x)* ≥ *P_min_*, where *P_min_* is the minimum probability density threshold). A consensus rule was adopted: an instance was considered out of domain if more than two of the four methods classified it as such. The number of out-of-domain instances for each dataset is presented in Table 3. Results indicate that none of the PhysChem-based datasets contained out-of-domain instances, while between one and four such instances were detected in the QuBiLs-MAS-based datasets (representing less than 0.4% of the total). This outcome demonstrates the high coverage of the models, which is significant because fewer out-of-domain instances imply greater versatility of the trained models. Finally, test datasets were refined to contain only in-domain instances. Representative fungicides captured within or without the applicability domain are shown in Figure 8.

### 2.7. External Validation of Optimized Models

**Figure 9:**
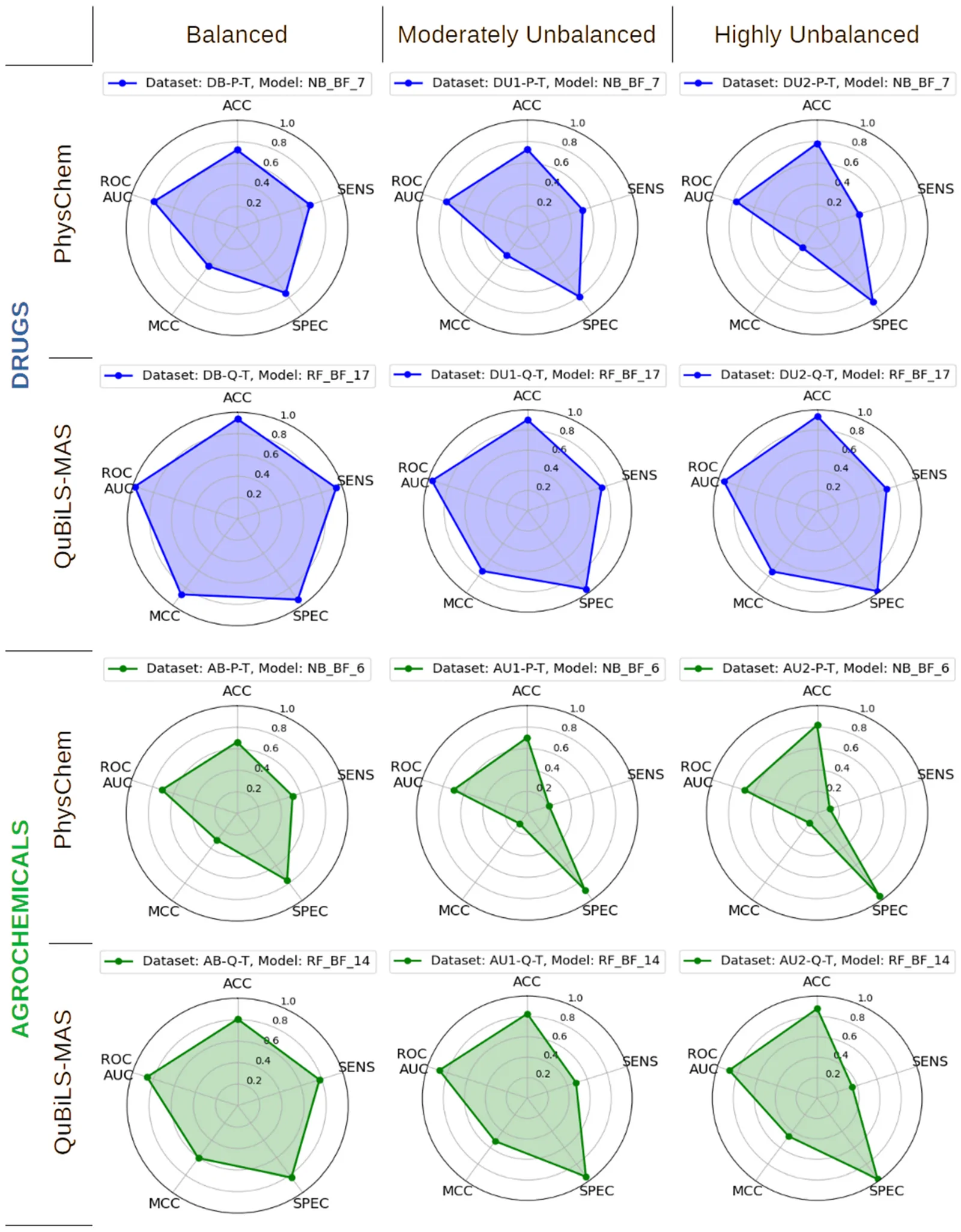
Results of External Validation of the 12 test datasets. Results were obtained through the ‘Supplied test set’ option in Weka, using each corresponding trained model. Drug-based datasets are shown in blue and agrochemical-based ones are shown in green.

The 12 refined test datasets were evaluated in Weka using their corresponding classifier methods and optimized trained models, and the resulting performance metrics are discussed (see Figure 9).

As before, almost balanced datasets consistently outperformed unbalanced ones: sensitivity (SENS) and Matthews’ correlation coefficient (MCC) decreased, specificity (SPEC) increased, and ROC-AUC was the least-affected parameter as the imbalance ratio grew. Models based on QuBiLs-MAS descriptors remain superior results compared to those derived from PhysChem descriptors across all metrics (see Supplementary Information S5). A multi-factor PerMANOVA using marginal effects confirmed that both factors significantly impact on this overall performance (*p* = 0.001), with the choice of the descriptor acting as the primary driver of variance (53.6%), followed closely by the dataset balance (21.7%) (see Supplementary Information S6). The best models were RF_BF_17, with ROC-AUC = 0.981, MCC = 0.872, and ACC = 0.937 in the dataset, and RF_BF_14, with ROC-AUC = 0.861, MCC = 0.605, and ACC = 0.806.

Owing to the origin of the negative-class instances, the agrochemical-associated model is considered stricter than the drug-associated model. This data origin also represents a statistically significant source of performance variance (21.3%, *p* = 0.001) (see Supplementary Information S6). Despite differences in numerical performance, both models are useful when combined in a positive consensus strategy, whereby a molecule is classified as positive only if both models agree. Such ensemble strategies improve prediction reliability [38,59], with the drug-based model offering higher sensitivity (fewer false negatives discarded) and the agrochemical-based model, due to its greater similarity to fungicides, providing stricter control by identifying residual false positives.

**Figure 10:**
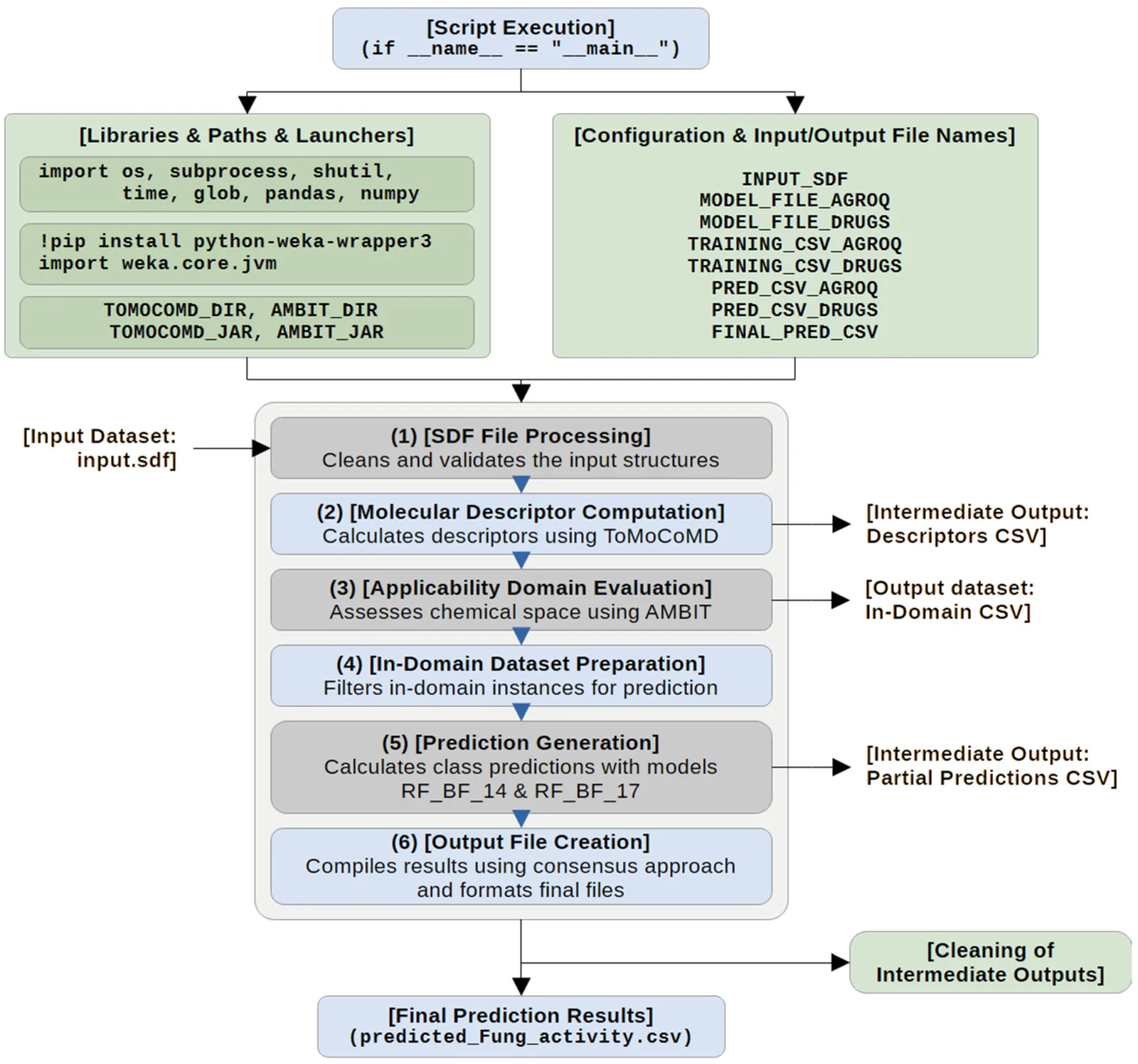
Scheme for Script Structure of FLiP-Z tool. Prediction results are obtained after providing the input.sdf file, which must contain the 3D optimized coordinates in SDF format of all molecules to be classified by FLiP-Z. ToMoCoMD.jar, Ambit.jar, RF_BF_14.model, RF_BF_17.model, and the corresponding training CSV datasets are supplied alongside the FLiP-Z Python script by permission.

Finally, based on the findings, the Python-based tool FLiP-Z was developed (see Section 3), which implements the RF_BF_17 and RF_BF_14 models to carry out the consensus approach outlined earlier (See Figure 10).

### 2.8. Case of Study: Prediction of Fungicide Likeness Using FLiP-Z

Filters were applied within ChEMBL to generate the molecular dataset that served as input for predicting fungicide likeness with the FLiP-Z tool. Compounds were selected according to three criteria: classification as small molecules, zero violations of the Rule of Five (#RO5), and a molecular weight below 900 Da. The filtered dataset was downloaded in CSV format from the ChEMBL web page, yielding 1,348,267 molecules. Data preprocessing was performed using RDKit, an open-source cheminformatics toolkit, and comprised several stages. First, molecules exhibiting high structural similarity (e.g., optical isomers, tautomers, others) were removed to reduce computational demand, resulting in the exclusion of approximately 70,000 entries. Subsequently, an additional 50,000 molecules were discarded through the systematic cleaning pipeline outlined in Table 1. The majority of these (99.9%) were eliminated due to duplication following SMILES standardization, while the remainder were excluded because they were inorganic compounds or mixtures; SMILES contained in the respective model’s training sets were also removed. Finally, during the conversion of SMILES to SDF format, some molecules were lost owing to unsuccessful generation of optimized three-dimensional molecular coordinates. The final curated dataset (Chembl_total) comprised 1,226,795 molecules in the SDF format.

**Figure 11:**
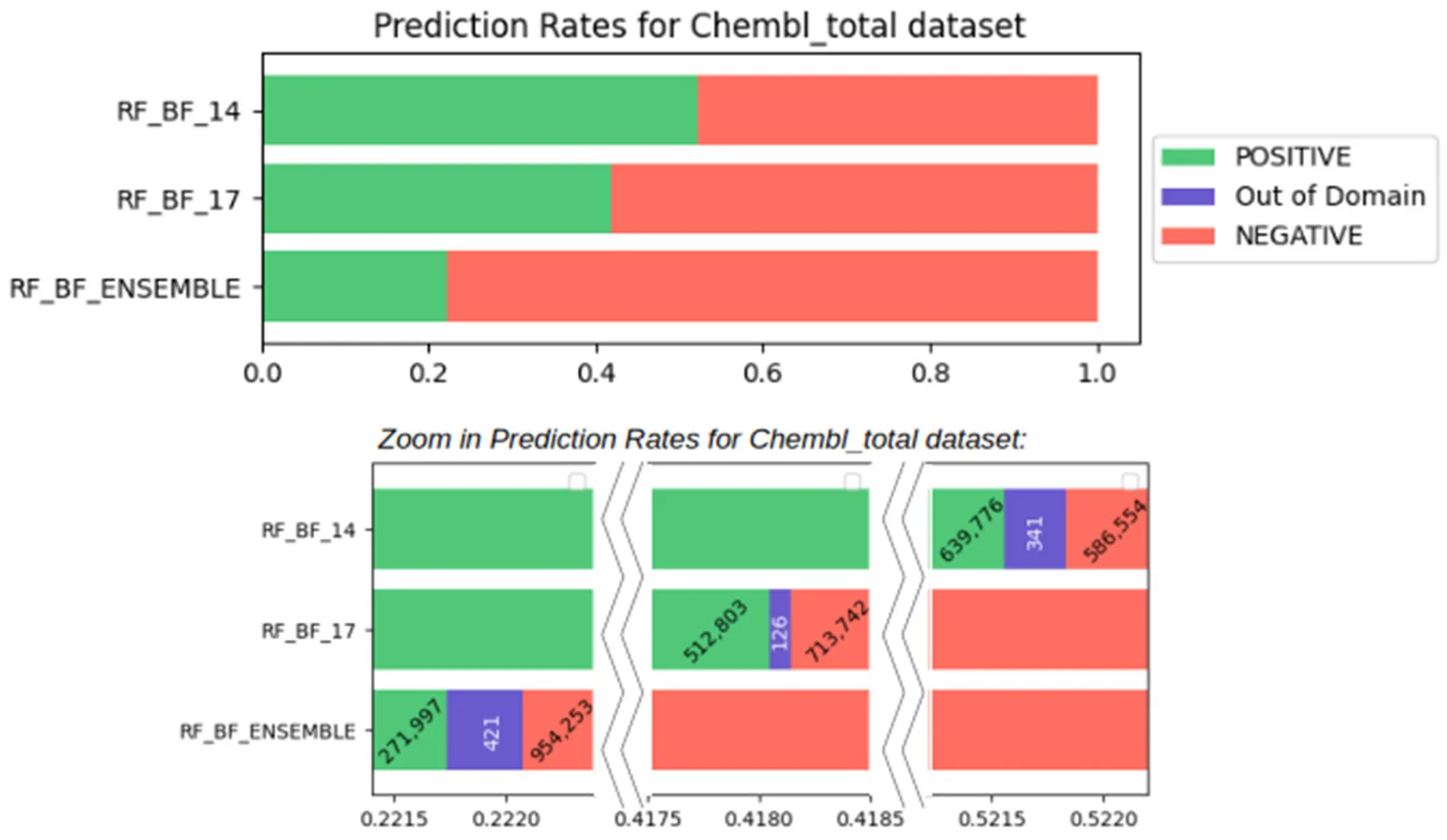
Prediction Rates of Fungicide-likeness for Chembl_total dataset using FLiP-Z. Prediction results for the input dataset of approximately 1.2 million molecules. The results illustrate the performance of individual models compared to the final ensembled model utilized in FLiP-Z. Additionally, three zoomed-in visualizations are provided to offer a clearer view of the out-of-domain instances.

After providing the input SDF file to the FLiP-Z Python-based tool (detailed instructions are available in the user’s guide under permission), the executable was run. The Python script performed the following tasks: (1) processing of the SDF file, (2) computation of molecular descriptors, (3) evaluation of the applicability domain, (4) preparation of datasets containing in-domain instances, (5) generation of predictions, and (6) creation of output files. Prediction results for the input dataset of approximately 1.2 million molecules are shown in Figure 11. While individual models predicted between 42% and 52% of instances as belonging to the positive class, the consensus approach ultimately identified about 22% (271,997 molecules) with positive fungicide-likeness according to both models. This high percentage reflects the models’ dependency on topological and physicochemical features rather than on specific bioactivity, in accordance with the nature of QuBiLS-MAS descriptors. Consequently, fungicide-likeness in the present work should be interpreted as a description of chemical similarity to known fungicides, whereas actual bioactivity remains to be experimentally tested.

It is important to note that only a very small fraction of instances was removed during the applicability domain evaluation (See Figure S1.6 in Supplementary Information S1). In total, 421 molecules were identified as out-of-domain (if a molecule falls outside the domain of one model, it is excluded from the dataset even if it lies within the domain of the second). This represents just 0.03% of the entire dataset, highlighting the broad coverage and versatility of the trained models implemented in FLiP-Z. Additionally, to assess independence between the two models, we computed pairwise agreement and correlation statistics (Cohen’s kappa, Cramér’s V, and Pearson correlation) on the approx. 1.2M molecules within the applicability domain of both classifiers. Agreement was at chance level (κ = 0.015; Cramér’s V = 0.015), with a 49.6% disagreement rate and negligible correlation between prediction scores (r = −0.020). This near-chance agreement indicates the two models do not produce systematically similar classifications, supporting their complementarity in the consensus strategy.

**Figure 12:**
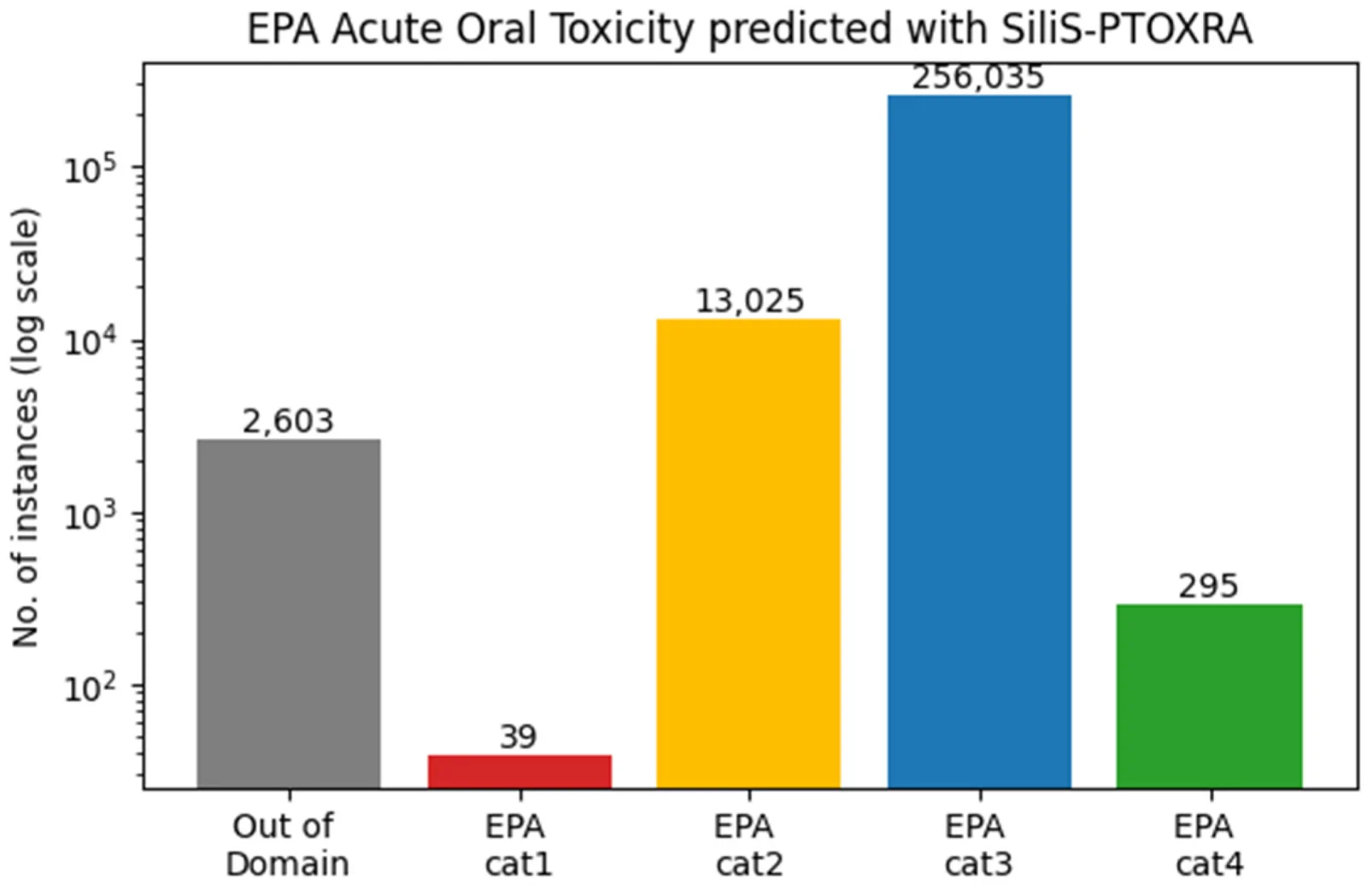
Predictions of Acute Oral Toxicity (AOT) of Positive Fungicide-like molecules. The predictions of AOT obtained with SiliS-PTOXRA on the positive dataset of approximately 270,000 fungicide-like molecules are illustrated in the figure. These predictions categorize instances into four toxicity categories as defined by the EPA [60]. The gray bar indicates instances that fall outside the domain, which the software was unable to predict.

Finally, to demonstrate how fungicide-likeness predictions can be integrated into a broader screening strategy, we performed a post-screening step using the software SiliS-PTOXRA v1.0 (https://tomocomd.com/apps/ptoxra, accessed on 20 August 2026) to predict the oral acute toxicity of the pool of candidate fungicides. This additional analysis further reduced the number of molecules while introducing an important dimension (toxicity considerations) that is critical when searching for promising lead compounds. As shown in Figure 12, the pool was narrowed to 295 candidate fungicides, with the added advantage that these molecules were predicted to exhibit low toxicity.

To provide scientific context for the 295 identified candidates, a structural analysis was conducted using RDKit. Although a significant number of molecules lack ring systems (30%), the remaining candidates yield a total of 112 distinct Murcko scaffolds, with the most prevalent being benzene derivatives (11%), cyclohexane-containing structures (4%), and two notably high-percentage scaffolds: C1CCOCC1 (3.7%) and C1CCC2(CC1)OOC1(CCCCC1)OO2 (3.4%). Additionally, 11 candidates share the same scaffold as 18 fungicides from the positive-class dataset. Among the candidates, 12.2% belong to chemical classes already represented by known fungicides (mainly carboxamides, as they are a very diverse fungicide family, but also some carbamates, pyrimidines, morpholines, and triazoles), supporting the biological plausibility of predictions. Remarkably, 149 candidates possess novel scaffolds that are not present in the BCPC compendium used to construct the positive training class, representing potentially unexplored chemical space for fungicide discovery. Prioritization for experimental validation should focus on a selection of candidates representing those groups with more prevalent scaffolds, as well as novel scaffolds relative to the training set. The dataset with all 295 candidates is the intellectual property of Zimeck C.L.

## 3. Materials and Methods

### 3.1. Dataset Construction

A well-constructed dataset is essential for developing robust machine-learning models. Since the goal of this study is to predict fungicide likeness, the dataset must have enough recognized fungicide molecules (positive class instances) that will be used for modeling and training processes. The model must be able to classify fungicides within a population that also contains an adequate number of molecules known to be non-fungicides (negative class instances). Here, we used two different sources for negative instances: (1) drugs obtained from the ChEMBL database, and (2) agrochemicals obtained from PubChem, both excluding fungicides already cataloged as positive instances. The two corresponding raw datasets comprised 486 positive and 15,591 negative molecules (drug-based, D0), and 486 positives along with 3022 negative molecules (agrochemical-based, A0). Then, a cleaning process was performed: molecules provided as SMILES strings were first standardized through cleanup, metal disconnection, normalization, deionization, and reionization. Duplicate compounds were removed based on canonical non-isomeric SMILES, while mixtures, inorganic molecules lacking carbon, organometallic compounds, and Markush or query structures were systematically excluded. For retained molecules, 2D coordinates were generated, and 3D conformers were constructed and optimized with the Universal Force Field (UFF). Curated datasets finished with 376 positive and 3728 negative instances (D1), and the same 376 positive along with 2681 negative instances (A1).

In addition to the construction of curated datasets containing confirmed positive and negative class molecules, other post-treated datasets were also constructed through a balancing process. Positive and negative class instances were balanced by applying sampling methods [61,62,63,64] based on unsupervised clustering selection, where physicochemical descriptors (described below) were employed for K-means clustering into 12 clusters, employing a fixed random seed to ensure reproducibility. Within each cluster, molecules were ranked and sampled to create the datasets.

### 3.2. Descriptors Calculation

Traditional physicochemical-based descriptors (PhysChem) used in drug design were first employed to evaluate performance. One-dimensional (1D) and two-dimensional (2D) descriptors such as molecular weight (MW), number of hydrogen bond donors (HBD) and acceptors (HBA), number or rotatable bonds (ROB), number of rings (Rings), number of oxygen (nO) or nitrogen (nN) atoms, octanol–water partition coefficient (AlogP), and topological polar surface area (TPSA) were selected according to previous studies [7] in which fungicide-likeness was estimated. Subsequently, 2.5D descriptors were calculated by using the software QuBiLs-MAS [36]. For these, SMILES representations of all molecules in the dataset were used to generate their three-dimensional structures via a UFF force field implemented in the Python library RDKit, after which the QuBiLs-MAS descriptors were calculated. Pearson correlation coefficients (r = 0.7) were applied to estimate descriptor correlations and collinearity, reducing the number of variables. Also, an analysis based on Shannon’s entropy was applied to eliminate descriptors with near-zero variance by using a cutoff of 60% of the maximum entropy value [65].

### 3.3. Models Selection Trough Internal Cross-Validation

Models were systematically constructed in Weka 3.8.6 [66] by combining a defined range of descriptors for each model with different meta-classification algorithms. Feature selection was performed by applying a wrapper-based subset evaluation scheme [67] inside each cross-validation fold to identify informative descriptor subsets for each dataset. Specifically, the WrapperSubsetEval evaluator was combined with multiple learning algorithms (Naive Bayes [NB], Logistic Regression [LOG], J48 decision tree [JT], Fisher’s Linear Discriminant Analysis [FLDA], Support Vector Machine [SMO], Random Forest [RF], and k-Nearest Neighbors [IBk]), and paired with four search strategies (BestFirst, GreedyStepwise, GeneticSearch, and PSO [Particle Swarm Optimization]). For IBk, the wrapper evaluation was configured with 10 neighbors and cross validation, while default settings were used for the remaining classifiers. BestFirst was executed with a bounded search configuration (depth and node limits), whereas the other search methods were run with default parameters. For each dataset, every classifier searched for a reduced feature set, and the resulting reduced datasets were exported as separate files and labeled according to the classifier, search method, and number of selected attributes. Through cross-validation, a strategy that is usually termed as internal validation and that provides a reasonable approximation of the prediction power of a model [68], the most accurate models (the top 10 or top 20, depending on the range of obtained accuracies) were pre-selected. Subsequently, each model was evaluated, with the seven base classifiers leading to the final selection of the best models to be used for each initial dataset and kind of descriptor.

### 3.4. Training in Selected Models

**Figure 13:**
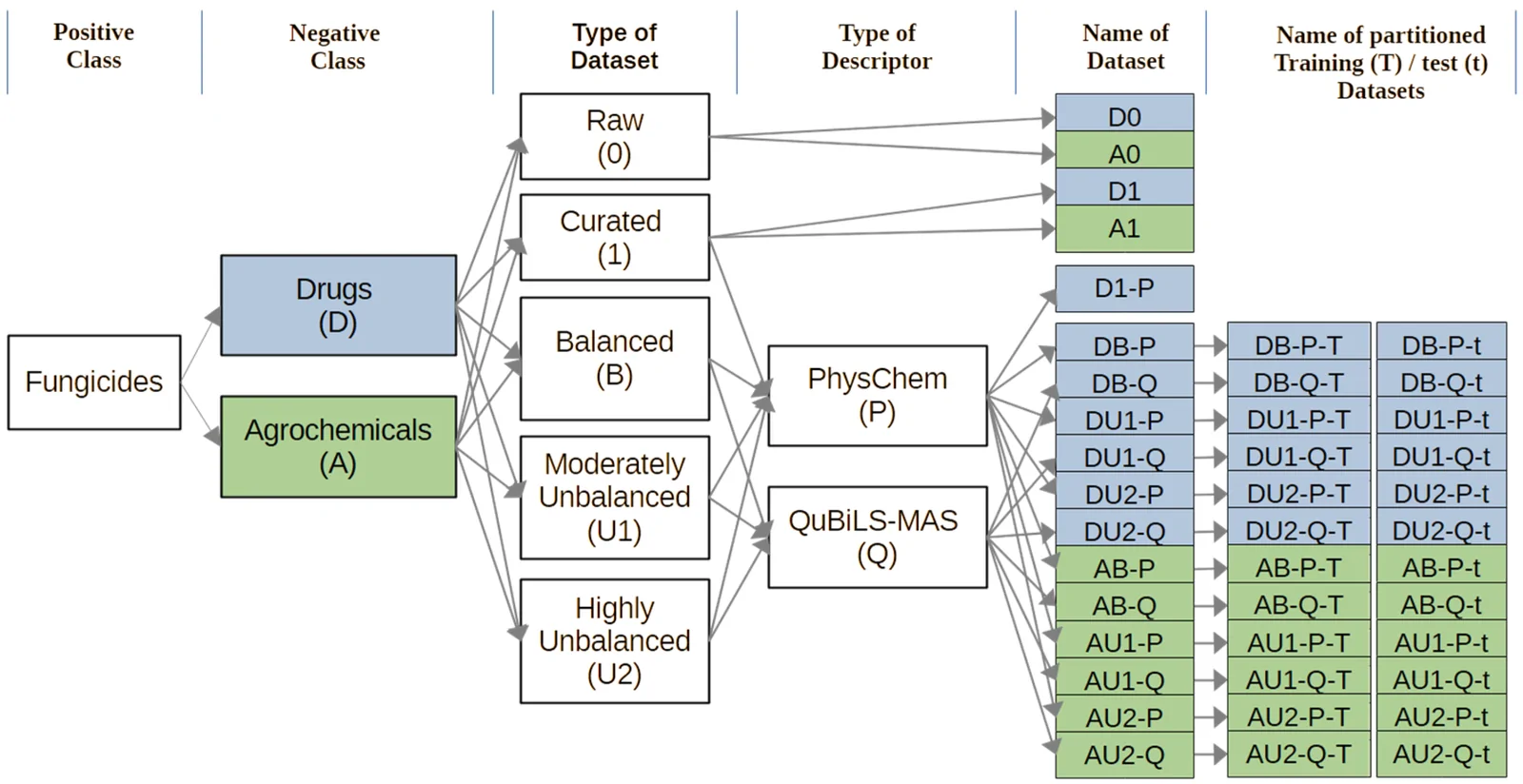
Detailed process for dataset construction across various variables. The acronyms for each variable are presented in parentheses, and each dataset is coded according to its respective combination of variables.

Once the candidate models were selected, they were trained using different types of datasets. These datasets were categorized according to the origin of their negative instances (derived either from general drugs or from non-fungicidal agrochemicals), the composition of positive and negative instances (balanced, moderately unbalanced, or highly unbalanced), and the type of descriptors employed (PhysChem or QuBiLs-MAS). These variations were introduced to systematically evaluate and compare model performance across different conditions (See Figure 13).

**Figure 14:**
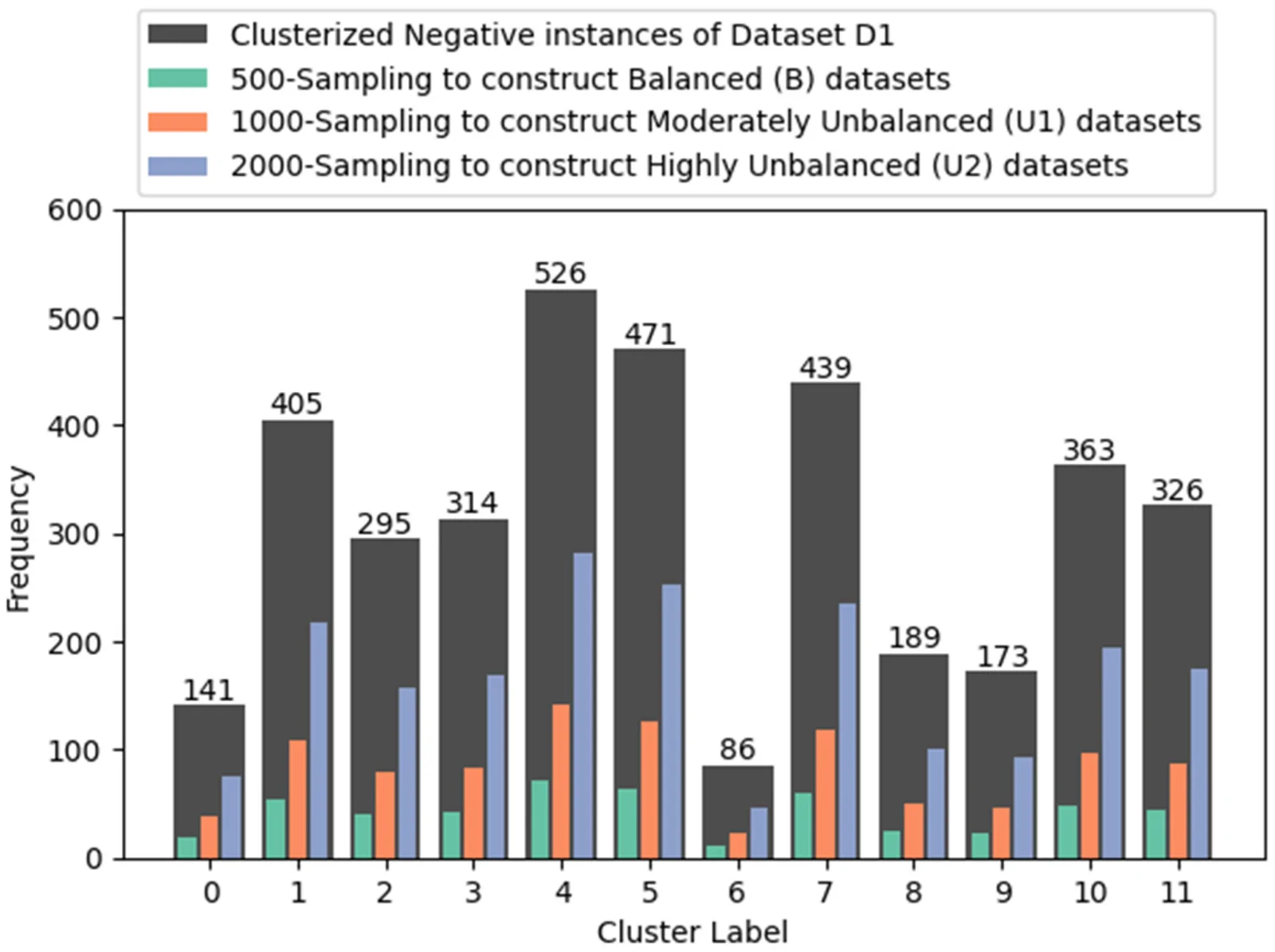
Construction of Derived Datasets with Varying Levels of Balance. This figure illustrates the construction of derived datasets from the drug-based curated dataset D1. The dataset D1 was clustered into 12 groups, and proportional amounts of instances were sampled from each cluster to create both balanced and unbalanced datasets. A similar process was applied to the agrochemical-based dataset A1 (see Figure S1.7 in Supplementary Information S1).

At this stage, it is important to describe the methodology used to obtain the three levels of balanced compositions of positive and negative instances. Across all datasets, positive instances were fully retained without subsampling, whereas negative instances were selected through clustering techniques [62,63,64] applied to the numerical descriptor values as described above. By preserving the relative proportions among the identified clusters during this process (see Figure 14), the diversity and representativeness of the negative class were maintained, regardless of the number of instances ultimately included.

Then, each of the resulting datasets, with their corresponding descriptors calculated, was partitioned using a 70:30 ratio to generate training and test sets. The test sets were reserved for performance evaluation after training. Although the partitioning was performed randomly, the randomization process was constrained by recommended stratification techniques [69] to preserve the distribution of positive and negative instances across both training and test subsets.

Finally, for each pair of training and test datasets, the corresponding selected models were trained by the software Weka 3.8.6 using the Random Forest classifier and default hyperparameters [-P 100 -I 100 -num-slots 1 -K 0 -M 1.0 -V 0.001 -S 1]: 100% of the training set size used for bagging, 100 decision trees (iterations), execution in 1 CPU core (slot), number of attributes per split set to 0 (default Weka’s formula K = int(log_2_(number of predictors) + 1)), minimum of 1 instance per leaf, a threshold of 0.001 as the minimum proportion of variance for a split to be considered, and a fixed random seed of 1 to ensure full reproducibility of results [66].

### 3.5. External Validation of Models

After training, validation of models using the reserved test datasets, usually termed as external validation [68], is of critical importance to evaluate the true predictive power of the model [70]. Test datasets were evaluated in Weka to obtain their respective performance metrics, including [71]:

- Accuracy (ACC), the proportion of correctly classified instances (true positives and true negatives) over the total number of instances;
- Sensitivity (SENS, also called Recall or True Positive Rate), the proportion of correctly identified positive instances relative to all actual positives;
- Specificity (SPEC, or True Negative Rate), the proportion of correctly identified negative instances relative to all actual negatives;
- Matthews’ Correlation Coefficient (MCC), a balanced measure of classification quality that accounts for true and false positives and negatives, ranging from –1 (total disagreement) to +1 (perfect prediction); and
- Area Under the Receiver Operating Characteristic Curve (ROC-AUC), a measure of the model’s ability to discriminate between classes, representing the probability that a randomly chosen positive instance is ranked higher than a randomly chosen negative instance.

Then, a multi-factor Permutational Analysis of Variance (PerMANOVA) was conducted using the marginal effects of terms (adonis2 function via the R vegan package version 2.7-5) to simultaneously evaluate the impact of dataset balance, negative class origin, and descriptor type on the overall performance. Given that the five performance metrics are inherently correlated (since they all depend on the same data, any alterations will impact on all of them) and represent different dimensions of a singular outcome, a multivariate approach was required. PerMANOVA was chosen because its permutation-based design does not require parametric error terms, making it ideal for deterministic computational data that lacks random experimental error.

### 3.6. Optimization of Models

According to Tropsha [70], once models that have passed internal and external validation have been selected, the next step is to perform the analysis of the applicability domain (AD) approach. Since being inside the AD according to a single method is not considered sufficient for accepting a prediction [55,56], the use of consensus methods has been strongly recommended [57]. Additionally, in the present work, a refinement task detecting descriptors with low relevance to the model was performed before executing this AD-based validation step, thereby obtaining the definitive performance metrics of the models. Also, for the best models, a detailed analysis of the descriptors was performed combining near-zero variance filtering, univariate relevance analysis based on mutual information and the area under the Mann–Whitney–derived curve, correlation-based redundancy removal (|r| ≥ 0.7), and multicollinearity control using variance inflation factors (VIF > 10) [47].

### 3.7. Development of the Python-Based Tool

#### FLiP-Z—Fungicide-Likeness Predictor by Zimeck

With the aim of developing a tool to facilitate the application of the present machine-learning models in the discovery of new fungicidal molecules, selected models were implemented in Python as a consensus strategy. This strategy predicts the fungicide-likeness of input molecules, classifying them as positive only when the models agree and the molecules fall within the applicability domain of the models. The Python script utilizes several derivative tools, including ToMoCoMD and its software QuBiLs-MAS, for calculating QuBiLs-MAS descriptors, as well as RDKit, Ambit Discovery, and Weka for performing specific tasks within the prediction pipeline. Although it was designed as a self-executable tool, it might require prior installation of the aforementioned libraries or launchers. The benefits of FLiP-Z include the capacity to perform predictions on large-scale datasets, as will be demonstrated in the case study presented herein, and the reliability of the model performance achieved through a rigorous and well-established validation methodology. Both the FLiP-Z tool and a user’s manual with instructions on its use and result interpretation are available upon request at flip-z@zimeck.com.ec.

### 3.8. Case of Study

The methodology described in the present case study provides an example of how the developed tool can be employed to perform screening tasks. After selecting and curating a database of interest, users provide the corresponding SDF data, and FLiP-Z executes the following operations: calculation of QuBiLs-MAS descriptors, verification of applicability domain compliance, and classification of molecules as positive or negative for fungicide-likeness. In this study, we incorporated an additional filtering step using acute oral toxicity predictions from another software included in ToMoCoMD: SiliS-PTOXRA, to demonstrate how the screening process can integrate supplementary criteria either before or after FLiP-Z predictions, thereby enhancing the relevance and utility of the results.

## 4. Conclusions

A curated framework for fungicide-likeness prediction was developed by integrating balanced datasets, topological molecular descriptors, and machine-learning techniques. The positive class contains 376 compounds, while two complementary negative classes were constructed from ChEMBL drugs (3728 molecules) and PubChem agrochemicals (2681 molecules). Dataset balancing substantially improved model robustness, increasing sensitivity and yielding higher MCC values despite a moderate reduction in accuracy. The use of QuBiLs-MAS 3D descriptors and Random Forest classifiers led to a marked performance improvement, with the optimized drug-based model (RF_BF_17) achieving ACC = 0.937, SENS = 0.943, SPEC = 0.933, MCC = 0.872, and ROC-AUC = 0.981, while the agrochemical-based model (RF_BF_14) reached ACC = 0.806, SENS = 0.778, SPEC = 0.828, MCC = 0.605, and ROC-AUC = 0.861, showing a stricter classification scenario due to higher structural similarity between classes in the latter. An excellent coverage was obtained in the applicability domain analysis, with less than 0.4% out-of-domain instances across all datasets. When applied to a large-scale virtual screening of about 1.2 million molecules using the FLiP-Z tool and a consensus strategy, 271,997 compounds (22.2%) were identified as potential fungicide-like candidates, which were further refined to 295 low-toxicity molecules through post-screening.

A limitation of the present study is that the positive training class comprised 376 fungicides, a relatively modest dataset that may not capture the full structural diversity of the antifungal chemical space, particularly for newer chemical classes. Due to the commercial status of FLiP-Z, dataset construction was restricted to publicly available databases; alternative sources that could have been used to increase the number of instances in the positive class were excluded, as their licenses restrict usage exclusively to research and explicitly forbid integration into commercial software or databases. Expanding the positive class as new fungicide registrations become available will be important for improving model coverage.

## Figures and Tables

**Table 1:** Pipeline implemented in Python for the cleaning process of raw datasets.

Step	Action	Criteria
(1)	Remove duplicates	by standardized canonical SMILES
(2)	Remove mixtures	molecules with >1 disconnected fragments
(3)	Remove inorganics	no-carbon
(4)	Remove organometallics	contains metals and carbon atoms
(5)	Remove Markush/query structures	query features
(6)	Remove sanitize failures	valence/aromaticity/etc.
(7)	Neutralize salts	de-ionize
(8)	Generate 2D and 3D	3D optimized with UFF (ETKDG + UFF)

**Table 2:** Number of instances and balance between positives and negatives of Datasets used for Training of selected models.

Dataset	Total Instances Balance: [Neg. | Pos.]	Training(T)/Test(t) Partition		Trained Model ^1^ (Training Dataset)
		T/t Ratio {T/t Instances}	T Balance: [Neg. | Pos.] t Balance: [Neg. | Pos.]	
DB-P	875 [499(−) | 376(+)]	70/30 {665/210}	T: [380(−) | 285(+)] t: [119(−) | 91(+)]	NB_BF_9 (DB-P-T)
DB-Q	875 [499(−) | 376(+)]	70/30 {665/209}	T: [379(−) | 286(+)] t: [119(−) | 90(+)]	RF_BF_17 (DB-Q-T)
DU1-P	1376 [1000(−) | 376(+)]	70/30 {1042/334}	T: [757(−) | 285(+)] t: [243(−) | 91(+)]	NB_BF_9 (DU1-P-T)
DU1-Q	1376 [1000(−) | 376(+)]	70/30 {1039/336}	T: [753(−) | 286(+)] t: [246(−) | 90(+)]	RF_BF_17 (DU1-Q-T)
DU2-P	2376 [2000(−) | 376(+)]	70/30 {1790/586}	T: [1505(−) | 285(+)] t: [495(−) | 91(+)]	NB_BF_9 (DU2-P-T)
DU2-Q	2376 [2000(−) | 376(+)]	70/30 {1787/586}	T: [1501(−) | 286(+)] t: [496(−) | 90(+)]	RF_BF_17 (DU2-Q-T)
AB-P	858 [482(−) | 376(+)]	70/30 {650/208}	T: [365(−) | 285(+)] t: [117(−) | 91(+)]	NB_BF_9 (AB-P-T)
AB-Q	858 [482(−) | 376(+)]	70/30 {651/207}	T: [366(−) | 285(+)] t: [116(−) | 91(+)]	RF_BF_15 (AB-Q-T)
AU1-P	1376 [1000 | 376(+)]	70/30 {1040/336}	T: [755(−) | 285(+)] t: [245(−) | 91(+)]	NB_BF_9 (AU1-P-T)
AU1-Q	1376 [1000 | 376(+)]	70/30 {1041/335}	T: [756(−) | 285(+)] t: [244(−) | 91(+)]	RF_BF_15 (AU1-Q-T)
AU2-P	2296 [1920 | 376(+)]	70/30 {1730/566}	T: [1445(−) | 285(+)] t: [475(−) | 91(+)]	NB_BF_9 (AU2-P-T)
AU2-Q	2296 [1920 | 376(+)]	70/30 {1730/564}	T: [1445(−) | 285(+)] t: [473(−) | 91(+)]	RF_BF_15 (AU2-Q-T)

^1^ Although trained models are labeled with similar names for simplicity, they all have different training, each of them associated with their respective database (specified in parenthesis).

**Table 3:** Number of out-of-domain instances found in each test dataset.

Drug-Based Datasets		Agrochemical-Based Datasets	
Evaluated Dataset	Number of Out-of-Domain Instances	Evaluated Dataset	Number of Out-of-Domain Instances
DB-P-t	none	AB-P-t	none
DU1-P-t	none	AU1-P-t	none
DU2-P-t	none	AU2-P-t	none
DB-Q-t	2	AB-Q-t	1
DU1-Q-t	4	AU1-Q-t	2
DU2-Q-t	3	AU2-Q-t	1

## Data Availability

The curated positive dataset of 376 fungicides (Table S1.1) and all descriptor values used for model training are openly available in Supplementary Information. The trained Weka model files (RF_BF_14.model and RF_BF_17.model) and SMILES for the 295 screened molecules are available upon request, as well as the complete FLiP-Z Python source code that is proprietary software owned by Zimeck C.L. and is not publicly available; access may be requested at flip-z@zimeck.com.ec subject to company approval. The authors acknowledge that this restriction limits full computational reproducibility of the screening results reported in Section 2.8; however, the trained models, descriptor definitions, and validation datasets are sufficient to independently reproduce all classification performance metrics reported in Section 2.4, Section 2.5, Section 2.6 and Section 2.7.

## Supplementary Materials

The following supporting information can be downloaded at: https://www.mdpi.com/article/10.3390/ijms27177562/s1.

## Abbreviations

The following abbreviations are used in this manuscript:

ACC	Accuracy
AD	Applicability domain
AOT	Acute oral toxicity
AUC univar	Univariate performance–area under the curve
BCPC	Compendium of Pesticide Common Names
CID	PubChem Compound ID
CSV	Comma-Separated Values format
EPA	Environmental Protection Agency
FLiP-Z	Fungicide Likeness Predictor by Zimeck
MCC	Matthews correlation coefficient
MI	Mutual information
NB	Naive Bayes
PhysChem	Group of nine selected physicochemical descriptors
QSAR	Quantitative Structure–Activity Relationship
QuBiLs	Quadratic, Bilinear and Linear Maps
QuBiLs-MAS	(1) acronym for QuBiLs descriptors based on graph–theoretic electronic-density matrices and atomic weightings, (2) software for calculation of QuBiLs-MAS descriptors (https://tomocomd.com/software/qubils-mas)
RF	Random Forest
ROC-AUC	Receiver operating characteristic–area under the curve
SDF	Structured Data File format
SENS	Sensitivity
SMILES	Simplified Molecular Input Line Entry System
SPEC	Specificity
ToMoCoMD	Shorten term referring to the framework ToMoCoMD-CARDD (https://tomocomd.com/) which contains various software and applications
UFF	Universal Force Field
USAN	United States Adopted Name
VIF	Variance inflation factor
