# Supplementary material for: FLiP-Z by Zimeck: A Python-Based Machine-Learning Tool for Predicting Fungicide-Likeness of Organic Molecules
Source: Int J Mol Sci. 2026 Aug 24;27(17):7562. doi: 10.3390/ijms27177562 (PMC13566103; doi:10.3390/ijms27177562)
Supplement: Supplementary file 1 [file ijms-27-07562-s001.zip › SupplementaryInformation_S1.pdf]

Supplementary Information S1:

Additional Figures and Tables

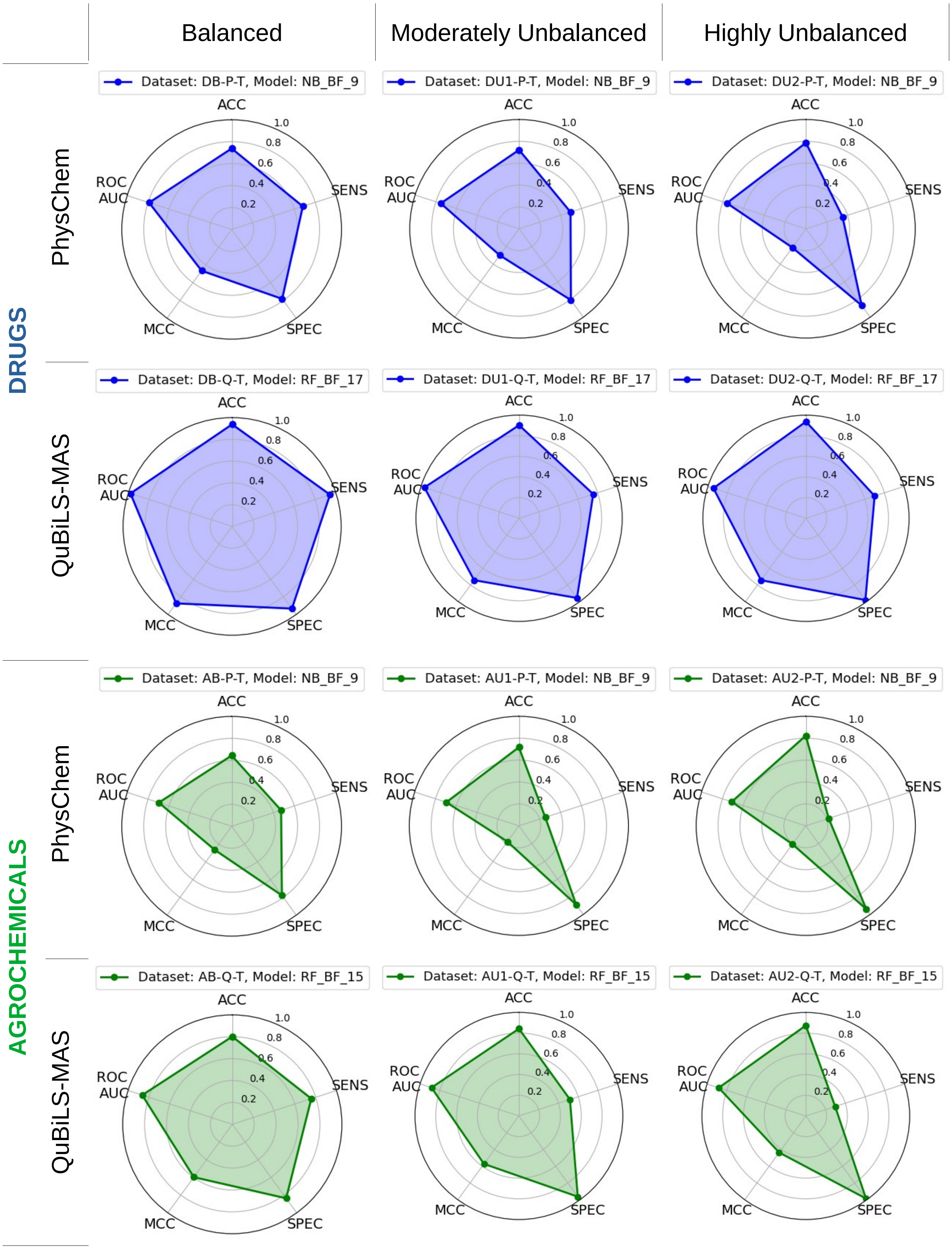

Figure S1.1 Results of External Validation of the twelve test datasets before Optimization. Results were obtained through the ‘Supplied test set’ option in Weka, using each corresponding trained model. Drug-based datasets are shown in blue and agrochemical-based ones are shown in green.

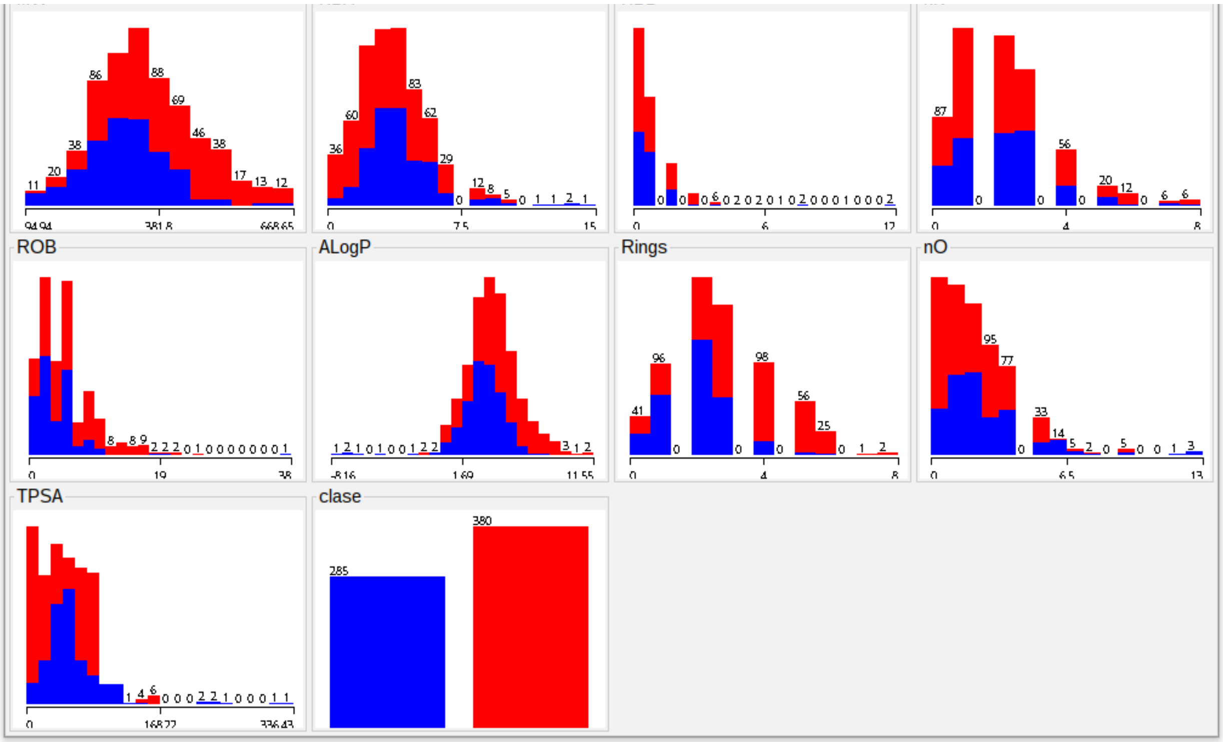

Figure S1.2 Graphical assessment of descriptor quality in DB-P-T. This is a capture taken from the software Weka illustrating the distribution curve of each descriptor. ‘clase’ refers to positive (Blue bars) and negative (Red bars) instances.

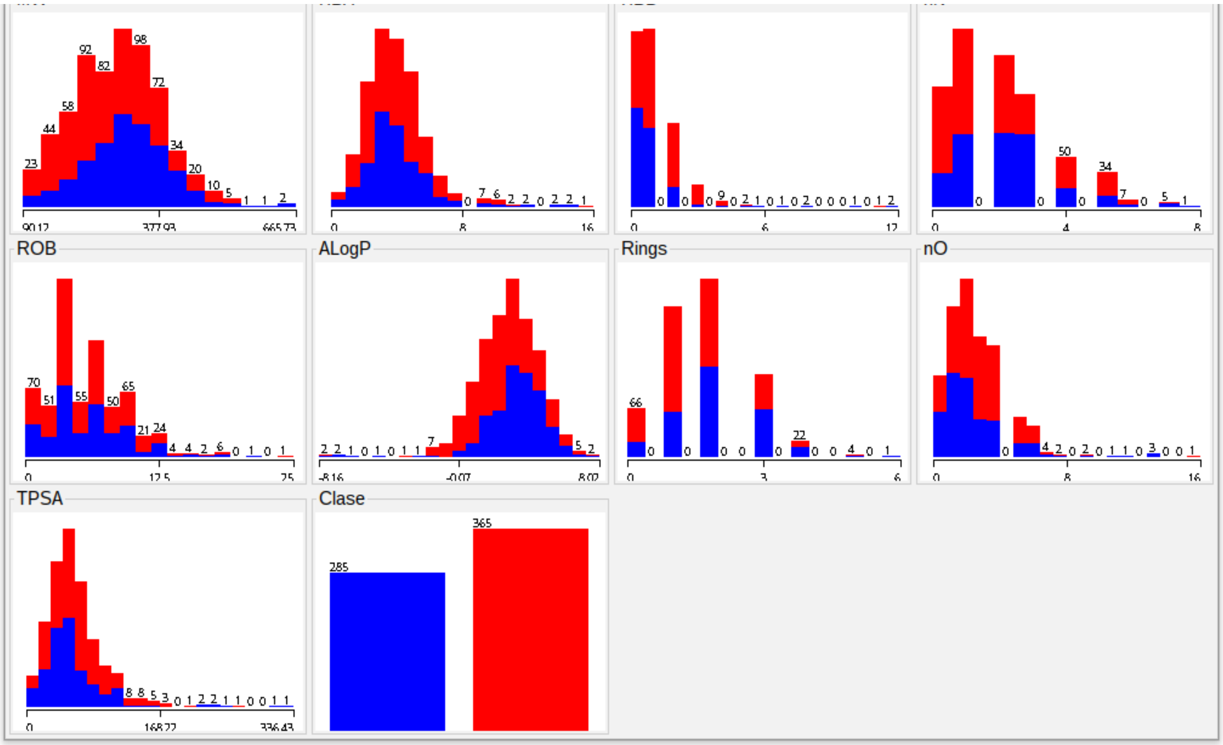

Figure S1.3 Graphical assessment of descriptor quality in AB-P-T. This is a capture taken from the software Weka illustrating the distribution curve of each descriptor. ‘Clase’ refers to positive (Blue bars) and negative (Red bars) instances.

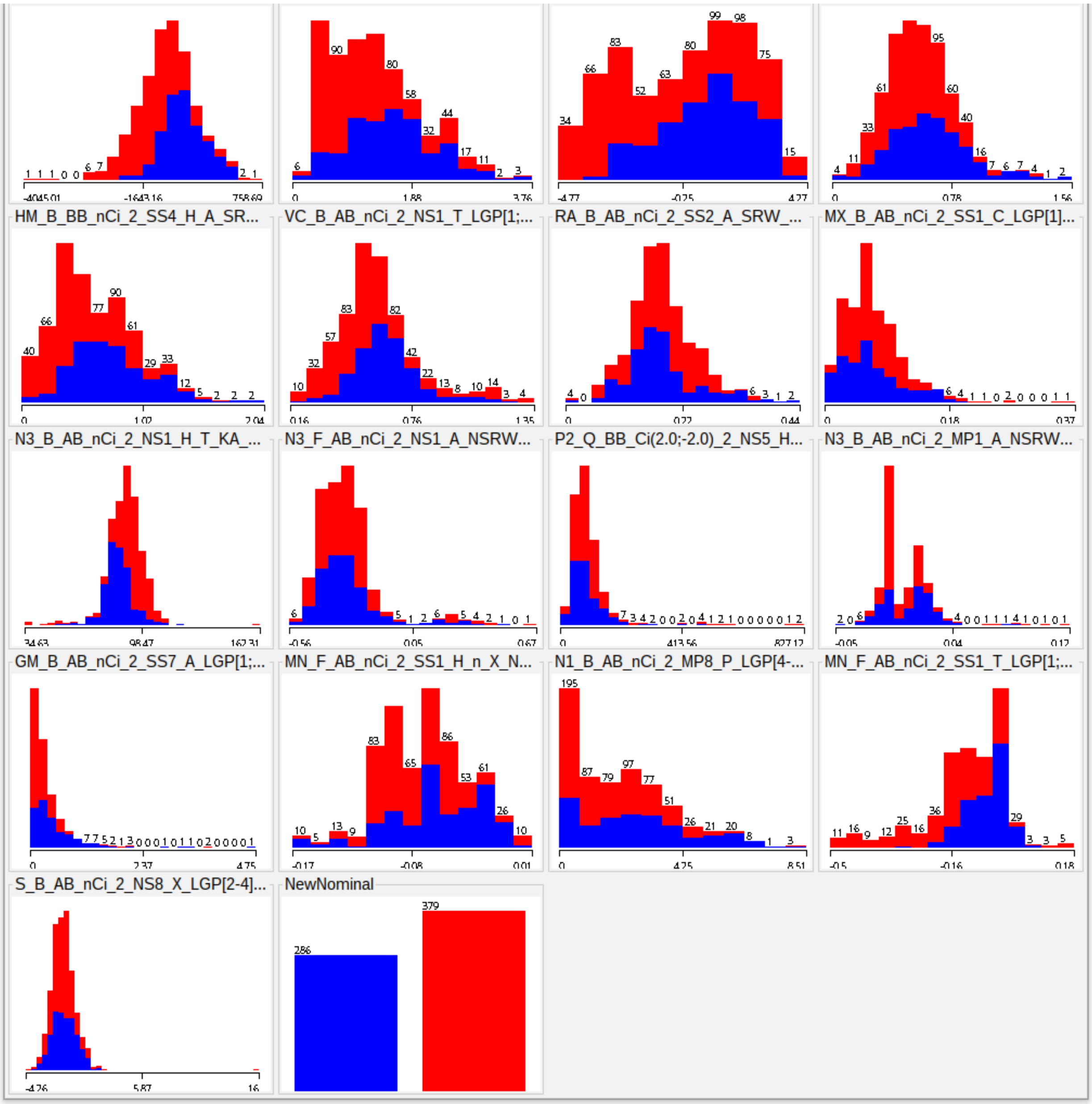

Figure S1.4 Graphical assessment of descriptor quality in DB-Q-T. This is a capture taken from the software Weka illustrating the distribution curve of each descriptor. 'NewNominal' refers to positive (Blue bars) and negative (Red bars) instances. Full names of descriptors are listed in Table S1.2

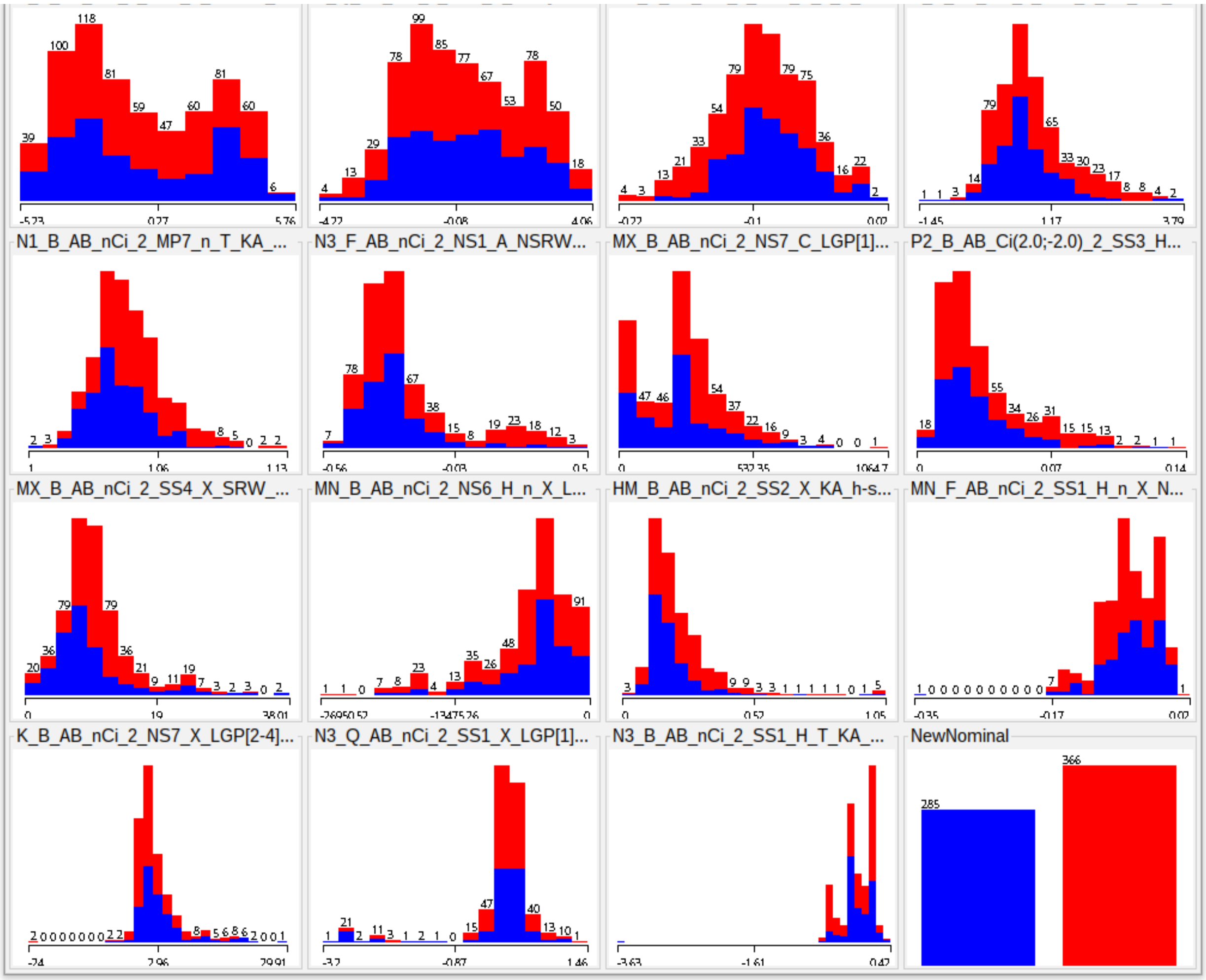

Figure S1.5 Graphical assessment of descriptor quality in AB-Q-T. This is a capture taken from the software Weka illustrating the distribution curve of each descriptor. 'NewNominal' refers to positive (Blue bars) and negative (Red bars) instances. Full names of descriptors are listed in Table S1.3

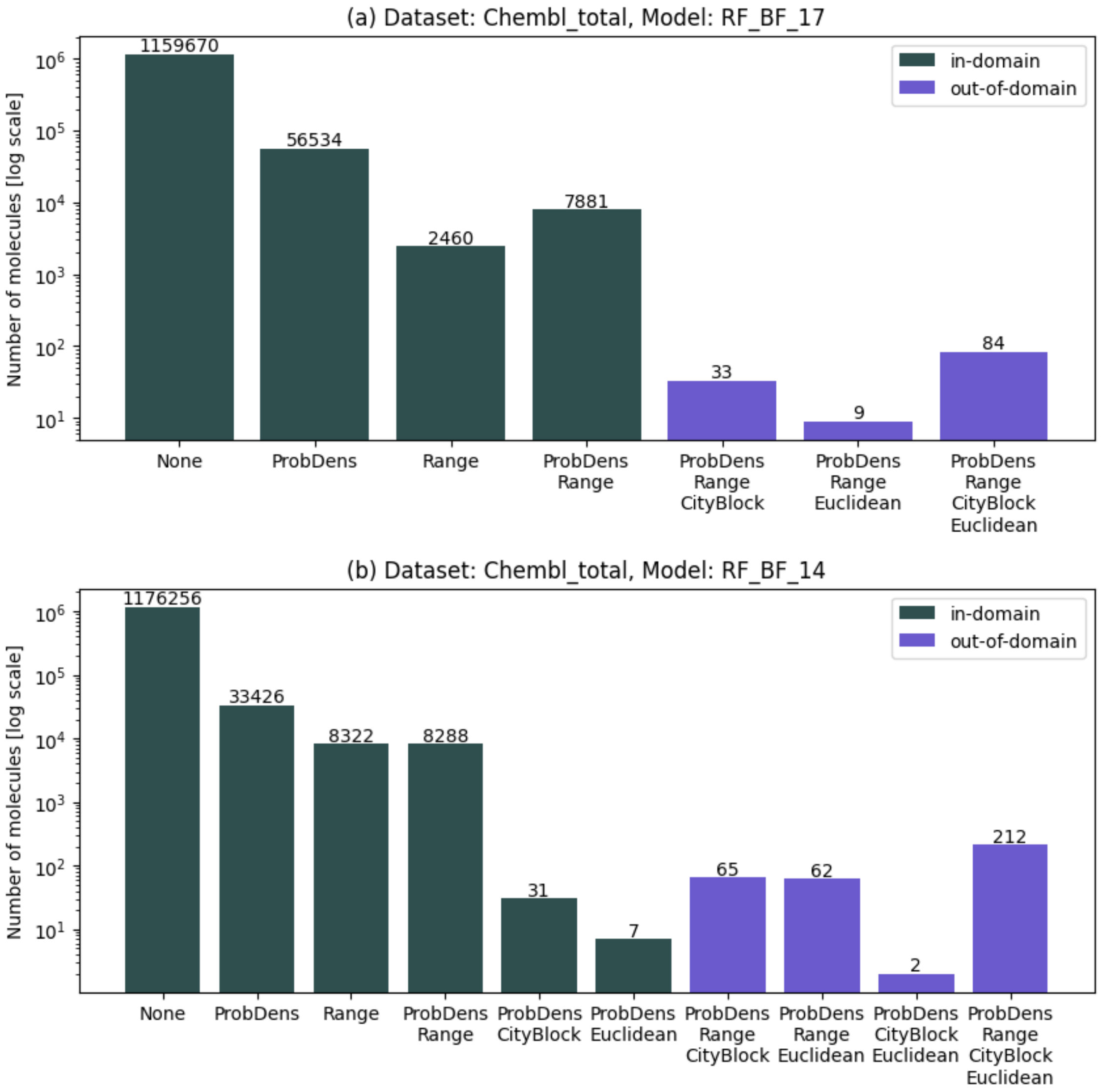

Figure S1.6. Distribution of per-method applicability domain results in the Chembl_total dataset. Number of molecules discarded by each applicability domain (AD) method. Molecules flagged by multiple methods simultaneously are grouped into distinct multi-method categories. In-domain and out-of-domain molecules according to the Consensus Rule are showed in different bar colors. (a) AD results related to model RF_BF_17; (b) AD results related to model RF_BF_14.

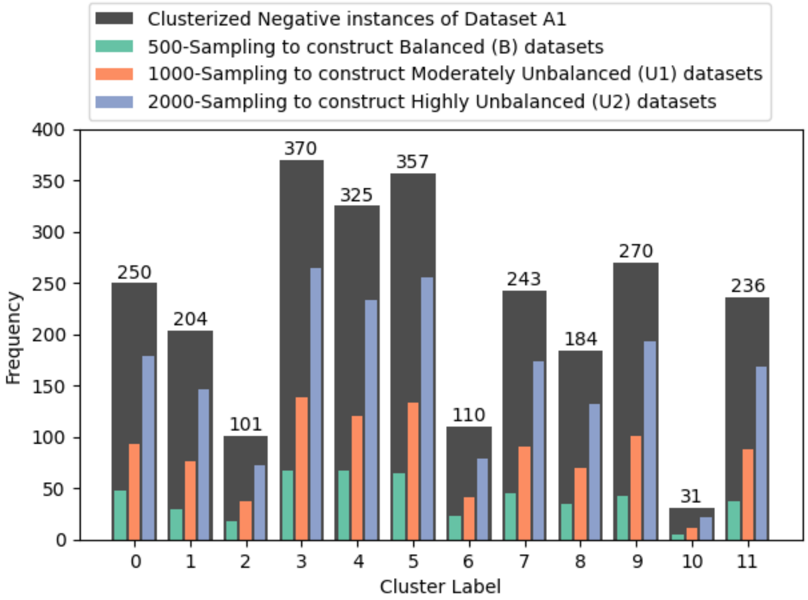

Figure S1.7. Construction of Derived Datasets with Varying Levels of Balance. This figure illustrates the construction of derived datasets from the agrochemical-based curated dataset A1. The dataset A1 was clustered into X groups, and proportional amounts of instances were sampled from each cluster to create both balanced and unbalanced datasets.

Table S1.1 Curated dataset of fungicides

No.	Name	PubChem CID	SMILES
1	butylamine	8007	CCCCN
2	dodicin	23280	CCCCCCCCCCCCNCCNCCNCC(=O)O
3	seboctylamine	93665	CCCCCCCCNCCNCCNCCCCCCCC
4	triazbutil	27760	CCCCn1cnnc1
5	benalaxyl	51369	COC(=O)C(C)N(C(=O)Cc1ccccc1)c1c(C)cccc1C
6	furalaxyl	42504	COC(=O)C(C)N(C(=O)c1ccco1)c1c(C)cccc1C
7	metalaxyl	42586	COCC(=O)N(c1c(C)cccc1C)C(C)C(=O)OC
8	cyprofuram	50590	O=C1OCCC1N(C(=O)C1CC1)c1cccc(Cl)c1
9	metsulfovax	88916	Cc1nc(C)c(C(=O)Nc2ccccc2)s1
10	pyracarbolid	32597	CC1=C(C(=O)Nc2ccccc2)CCCO1
11	flusulfamide	86268	O=[N+]([O-])c1ccc(NS(=O)(=O)c2ccc(Cl)c(C(F)(F)F)c2)c(Cl)c1
12	tolnifanide	11703132	CCN(c1ccc(Cl)cc1[N+](=O)[O-])S(=O)(=O)c1ccc(C)cc1
13	fenfuram	90590	Cc1occc1C(=O)Nc1ccccc1
14	furcarbanil	34281	Cc1cc(C(=O)Nc2ccccc2)c(C)o1
15	methfuroxam	34313	Cc1oc(C)c(C(=O)Nc2ccccc2)c1C
16	fenhexamid	213031	CC1(C(=O)Nc2ccc(O)c(Cl)c2Cl)CCCCC1
17	benodanil	27195	O=C(Nc1ccccc1)c1ccccc1I
18	flufenoxadiazam	135370838	O=C(Nc1ccccc1F)c1ccc(-c2noc(C(F)(F)F)n2)cc1
19	flutolanil	47898	CC(C)Oc1cccc(NC(=O)c2ccccc2C(F)(F)F)c1
20	mebenil	23466	Cc1ccccc1C(=O)Nc1ccccc1
21	mepronil	41632	Cc1ccccc1C(=O)Nc1cccc(OC(C)C)c1
22	salicylanilide	6872	O=C(Nc1ccccc1)c1ccccc1O
23	benzohydroxamic acid	10313	O=C(NO)c1ccccc1
24	flumetover	443033	CCN(C)C(=O)c1ccc(C(F)(F)F)cc1-c1ccc(OC)c(OC)c1
25	fluopicolide	11159021	O=C(NCc1ncc(C(F)(F)F)cc1Cl)c1c(Cl)cccc1Cl
26	fluopimomide	72734664	COc1c(F)c(F)c(C(=O)NCc2ncc(C(F)(F)F)cc2Cl)c(F)c1F
27	tioxymid	20293316	COc1c(C)cc(N=C=S)cc1C(=O)N(C)C
28	trichlamide	115078	CCCCOC(NC(=O)c1ccccc1O)C(Cl)(Cl)Cl
29	zarilamid	13455941	CCOC(C#N)NC(=O)c1ccc(Cl)cc1
30	zoxamide	122087	CCC(C)(NC(=O)c1cc(Cl)c(C)c(Cl)c1)C(=O)CCl
31	fluopyram	11158353	O=C(NCCc1ncc(C(F)(F)F)cc1Cl)c1ccccc1C(F)(F)F
32	diclocymet	11527333	CC(NC(=O)C(C#N)C(C)(C)C)c1ccc(Cl)cc1Cl
33	dimethomorph	86298	COc1ccc(C(=CC(=O)N2CCOCC2)c2ccc(Cl)cc2)cc1OC
34	flumorph	20056423	COc1ccc(C(=CC(=O)N2CCOCC2)c2ccc(F)cc2)cc1OC
35	cymoxanil	5364079	CCNC(=O)NC(=O)C(C#N)=NOC
36	carpropamid	153847	CCC1(C(=O)NC(C)c2ccc(Cl)cc2)C(C)C1(Cl)Cl
37	cyclafuramid	93178	Cc1cc(C(=O)NC2CCCCC2)c(C)o1
38	furmecyclox	43359	CON(C(=O)c1cc(C)oc1C)C1CCCCC1
39	mandipropamid	11292824	C#CCOc1ccc(CCNC(=O)C(OCC#C)c2ccc(Cl)cc2)cc1OC
40	carboxin	21307	CC1=C(C(=O)Nc2ccccc2)SCCO1
41	oxycarboxin	21330	CC1=C(C(=O)Nc2ccccc2)S(=O)(=O)CCO1
42	cyflufenamid	135515530	O=C(Cc1ccccc1)NC(=NOCC1CC1)c1c(C(F)(F)F)ccc(F)c1F
43	isofetamid	71657865	Cc1cc(OC(C)C)ccc1C(=O)C(C)(C)NC(=O)c1sccc1C
44	fenoxanil	11262655	CC(Oc1ccc(Cl)cc1Cl)C(=O)NC(C)(C#N)C(C)C
45	pyraziflumid	71679243	O=C(Nc1ccccc1-c1ccc(F)c(F)c1)c1nccnc1C(F)(F)F
46	benzovindiflupyr	51347655	Cn1cc(C(=O)Nc2cccc3c2C2CCC3C2=C(Cl)Cl)c(C(F)F)n1
47	bixafen	11434448	Cn1cc(C(=O)Nc2ccc(F)cc2-c2ccc(Cl)c(Cl)c2)c(C(F)F)n1

48	enprocymid	170102825	C=C(CN(C(=O)c1c(C(F)F)nn(C)c1F)C1CC1)c1ccc(Cl)cc1Cl
49	flubeneteram	123679328	Cn1cc(C(=O)Nc2ccccc2Oc2ccc(C(F)(F)F)cc2Cl)c(C(F)F)n1
50	fluindapyr	57343740	CC1CC(C)(C)c2c(F)ccc(NC(=O)c3cn(C)nc3C(F)F)c21
51	fluxapyroxad	16095400	Cn1cc(C(=O)Nc2ccccc2-c2cc(F)c(F)c(F)c2)c(C(F)F)n1
52	fenopyramid	148717219	Cn1cc(C(=O)Nc2ccccc2Oc2ccc(C(F)(F)F)cc2C(F)(F)F)c(C(F)F)n1
53	furametpyr	3083543	Cc1nn(C)c(Cl)c1C(=O)Nc1cccc2c1C(C)OC2(C)C
54	inpyrfluxam	70652048	CC1CC(C)(C)c2cccc(NC(=O)c3cn(C)nc3C(F)F)c21
55	isoflucypram	66553206	CC(C)c1ccc(Cl)cc1CN(C(=O)c1c(C(F)F)nn(C)c1F)C1CC1
56	isopyrazam	25271089	CC(C)C1C2CCC1c1c(NC(=O)c3cn(C)nc3C(F)F)cccc12
57	penflufen	11674113	Cc1nn(C)c(F)c1C(=O)Nc1ccccc1C(C)CC(C)C
58	penthiopyrad	11388558	CC(C)CC(C)c1sccc1NC(=O)c1cn(C)nc1C(F)(F)F
59	pydiflumetofen	56933411	CON(C(=O)c1cn(C)nc1C(F)F)C(C)Cc1c(Cl)cc(Cl)cc1Cl
60	pyrapropoyne	131698386	CC(C)ON=C(CNC(=O)c1cn(C)nc1C(F)F)c1ccc(C#CC2CC2)cc1Cl
61	sedaxane	11688533	Cn1cc(C(=O)Nc2ccccc2C2CC2C2CC2)c(C(F)F)n1
62	tolfenpyrad	10110536	CCc1nn(C)c(C(=O)NCc2ccc(Oc3ccc(C)cc3)cc2)c1Cl
63	boscalid	213013	O=C(Nc1ccccc1-c1ccc(Cl)cc1)c1cccnc1Cl
64	cyclobutrifluram	71766128	O=C(NC1CCC1c1ccc(Cl)cc1Cl)c1cccnc1C(F)(F)F
65	fenpicoxamid	51039126	COc1ccnc(C(=O)NC2COC(=O)C(Cc3ccccc3)C(OC(=O)C(C)C)C(C)OC2=O)c1OCOC(=O)C(C)C
66	florylpicoxamid	121463656	COc1ccnc(C(=O)NC(C)C(=O)OC(C)C(c2ccc(F)cc2)c2ccc(F)cc2)c1OC(C)=O
67	metarylpicoxamid	139473168	CCC(=O)Oc1c(OC)ccnc1C(=O)NC(C)C(=O)OC(C)C(C)c1ccccc1C
68	dichlofluanid	14145	CN(C)S(=O)(=O)N(SC(F)(Cl)Cl)c1ccccc1
69	tolylfluanid	12898	Cc1ccc(N(SC(F)(Cl)Cl)S(=O)(=O)N(C)C)cc1
70	isotianil	9796266	N#Cc1ccccc1NC(=O)c1snc(Cl)c1Cl
71	tiadinil	2804318	Cc1ccc(NC(=O)c2snnc2C)cc1Cl
72	ethaboxam	11174640	CCNc1nc(CC)c(C(=O)NC(C#N)c2cccs2)s1
73	thifluzamide	86389	Cc1nc(C(F)(F)F)c(C(=O)Nc2c(Br)cc(OC(F)(F)F)cc2Br)s1
74	silthiofam	9881821	C=CCNC(=O)c1c([Si](C)(C)C)sc(C)c1C
75	benthiavalicarb	20593234	CC(NC(=O)C(NC(=O)O)C(C)C)c1nc2ccc(F)cc2s1
76	iprovalicarb	10958189	Cc1ccc(C(C)NC(=O)C(NC(=O)OC(C)C)C(C)C)cc1
77	valifenalate	11338509	COC(=O)CC(NC(=O)C(NC(=O)OC(C)C)C(C)C)c1ccc(Cl)cc1
78	propamidine	64949	N=C(N)c1ccc(OCCCOc2ccc(C(=N)N)cc2)cc1
79	benzamacril	44145118	CN(C=C(C#N)C(=O)O)Cc1ccccc1
80	phenamacril	66655715	CCOC(=O)C(C#N)=C(N)c1ccccc1
81	fenpyrazamine	11493665	C=CCSC(=O)n1c(N)c(-c2ccccc2C)c(=O)n1C(C)C
82	cycloheximide	6197	CC1CC(C)C(=O)C(C(O)CC2CC(=O)NC(=O)C2)C1
83	griseofulvin	441140	COC1=CC(=O)CC(C)C12Oc1c(Cl)c(OC)cc(OC)c1C2=O
84	ningnanmycin	44588235	CNCC(=O)NC(CO)C(=O)NC1C(C(N)=O)OC(n2ccc(N)nc2=O)C(O)C1O
85	blasticidin-S	170012	CN(CCC(N)CC(=O)NC1C=CC(n2ccc(N)nc2=O)OC1C(=O)O)C(=N)N
86	streptomycin	19649	CNC1C(OC2C(OC3C(O)C(O)C(N=C(N)N)C(O)C3N=C(N)N)OC(C)C2(O)C=O)OC(CO)C(O)C1O
87	validamycin	443629	OCC1=CC(NC2CC(CO)C(OC3OC(CO)C(O)C(O)C3O)C(O)C2O)C(O)C(O)C1O
88	kasugamycin	65174	CC1OC(OC2C(O)C(O)C(O)C(O)C2O)C(N)CC1N=C(N)C(=O)O
89	aureofungin A	86278668	CNc1ccc(C(=O)CC(O)CCC(C)C2OC(=O)CC(O)CC(O)CC(O)CC(O)CC(O)CC(O)CC3(O)CC(O)C(C(=O)O)C(CC(OC4OC

			(C)C(O)C(N)C4O)C=CC=CC=CC=CC=CC=CC=CC2C)O3)cc1
90	aureofungin B	86278667	CC=CC=CC(=O)CC(O)CCC(C)C1OC(=O)CC(O)CC(O)CC(O)CC(O)CC(O)CC(O)CC2(O)CC(O)C(C(=O)O)C(CC(OC3OC(C)C(O)C(N)C3O)C=CC=CC=CC=CC=CC=CC1C)O2
91	natamycin	5284447	CC1CC=CC=CC=CC=CC(OC2OC(C)C(O)C(N)C2O)CC2OC(O)(CC(O)CC3OC3C=CC(=O)O1)CC(O)C2C(=O)O
92	biphenyl	7095	c1ccc(-c2ccccc2)cc1
93	chloroneb	17581	COc1cc(Cl)c(OC)cc1Cl
94	dicloran	7430	Nc1c(Cl)cc([N+](=O)[O-])cc1Cl
95	diphenylamine	11487	c1ccc(Nc2ccccc2)cc1
96	quintozene	6720	O=[N+]([O-])c1c(Cl)c(Cl)c(Cl)c(Cl)c1Cl
97	tecnazene	8330	O=[N+]([O-])c1c(Cl)c(Cl)cc(Cl)c1Cl
98	metrafenone	6451057	COc1cc(C)c(C(=O)c2c(OC)ccc(Br)c2C)c(OC)c1OC
99	pyriofenone	23082663	COc1cc(C)c(C(=O)c2c(OC)ncc(Cl)c2C)c(OC)c1OC
100	climbazole	37907	CC(C)(C)C(=O)C(Oc1ccc(Cl)cc1)n1ccnc1
101	clotrimazole	2812	Clc1ccccc1C(c1ccccc1)(c1ccccc1)n1ccnc1
102	fenapanil	43523	CCCCC(C#N)(Cn1ccnc1)c1ccccc1
103	imazalil	37175	C=CCOC(Cn1ccnc1)c1ccc(Cl)cc1Cl
104	oxpoconazole	11348992	CC1(C)COC(C)(CCCc2ccc(Cl)cc2)N1C(=O)n1ccnc1
105	pefurazoate	93532	C=CCCCOC(=O)C(CC)N(Cc1ccco1)C(=O)n1ccnc1
106	prochloraz	73665	CCCN(CCOc1c(Cl)cc(Cl)cc1Cl)C(=O)n1ccnc1
107	triflumizole	91699	CCCOCC(=Nc1ccc(Cl)cc1C(F)(F)F)n1ccnc1
108	viniconazole	2880	C=C(c1ccccc1OCc1cccc(Cl)c1)n1ccnc1
109	azaconazole	43233	Clc1ccc(C2(Cn3cncn3)OCCO2)c(Cl)c1
110	bitertanol	91656	CC(C)(C)C(O)C(Oc1ccc(-c2ccccc2)cc1)n1cncn1
111	bromuconazole	3444	Clc1ccc(C2(Cn3cncn3)CC(Br)CO2)c(Cl)c1
112	carmeconazole	142588676	COC(=O)C(O)(Cn1cncn1)c1ccc(Oc2ccc(Cl)cc2)cc1Cl
113	cyproconazole	86132	CC(C1CC1)C(O)(Cn1cncn1)c1ccc(Cl)cc1
114	diclobutrazol	53309	CC(C)(C)C(O)C(Cc1ccc(Cl)cc1Cl)n1cncn1
115	difenoconazole	86173	CC1COC(Cn2cncn2)(c2ccc(Oc3ccc(Cl)cc3)cc2Cl)O1
116	diniconazole	6436605	CC(C)(C)C(O)C(=Cc1ccc(Cl)cc1Cl)n1cncn1
117	epoxiconazole	107901	Fc1ccc(C2(Cn3cncn3)OC2c2ccccc2Cl)cc1
118	etaconazole	91673	CCC1COC(Cn2cncn2)(c2ccc(Cl)cc2Cl)O1
119	fenbuconazole	86138	N#CC(CCc1ccc(Cl)cc1)(Cn1cncn1)c1ccccc1
120	fluotrimazole	91600	FC(F)(F)c1cccc(C(c2ccccc2)(c2ccccc2)n2cncn2)c1
121	fluquinconazole	86417	O=c1c2cc(F)ccc2nc(-n2cncn2)n1-c1ccc(Cl)cc1Cl
122	flusilazole	73675	C[Si](Cn1cncn1)(c1ccc(F)cc1)c1ccc(F)cc1
123	flutriafol	91727	OC(Cn1cncn1)(c1ccc(F)cc1)c1ccccc1F
124	furconazole	188694	FC(F)(F)COC1CCC(Cn2cncn2)(c2ccc(Cl)cc2Cl)O1
125	hexaconazole	66461	CCCCC(O)(Cn1cncn1)c1ccc(Cl)cc1Cl
126	huanjunzuo	13577008	OC1(c2ccc(Cl)cc2)CCCCCC1n1cncn1
127	imibenconazole	93483	Clc1ccc(CSC(Cn2cncn2)=Nc2ccc(Cl)cc2Cl)cc1
128	ipconazole	86211	CC(C)C1CCC(Cc2ccc(Cl)cc2)C1(O)Cn1cncn1
129	ipfentrifluconazole	71230673	CC(C)C(O)(Cn1cncn1)c1ccc(Oc2ccc(Cl)cc2)cc1C(F)(F)F
130	mefentrifluconazole	71230671	CC(O)(Cn1cncn1)c1ccc(Oc2ccc(Cl)cc2)cc1C(F)(F)F
131	metconazole	86210	CC1(C)CCC(Cc2ccc(Cl)cc2)C1(O)Cn1cncn1
132	myclobutanil	6336	CCCCC(C#N)(Cn1cncn1)c1ccc(Cl)cc1
133	penconazole	91693	CCCC(Cn1cncn1)c1ccc(Cl)cc1Cl
134	propiconazole	43234	CCCC1COC(Cn2cncn2)(c2ccc(Cl)cc2Cl)O1
135	quinconazole	190636	O=c1c2ccccc2nc(-n2cncn2)n1-c1ccc(Cl)cc1Cl
136	simeconazole	10085783	C[Si](C)(C)CC(O)(Cn1cncn1)c1ccc(F)cc1
137	tebuconazole	86102	CC(C)(C)C(O)(CCc1ccc(Cl)cc1)Cn1cncn1

138	tetraconazole	80277	FC(F)C(F)(F)OCC(Cn1cncn1)c1ccc(Cl)cc1Cl
139	triadimefon	39385	CC(C)(C)C(=O)C(Oc1ccc(Cl)cc1)n1cncn1
140	triadimenol	41368	CC(C)(C)C(O)C(Oc1ccc(Cl)cc1)n1cncn1
141	triticonazole	6537961	CC1(C)CCC(=Cc2ccc(Cl)cc2)C1(O)Cn1cncn1
142	uniconazole	6436604	CC(C)(C)C(O)C(=Cc1ccc(Cl)cc1)n1cncn1
143	fluoxytioconazole	122704770	N#Cc1ccc(Oc2ccc(C(F)(F)C(O)(Cn3[nH]cnc3=S)c3ccc(F)cc3F)nc2)cc1
144	prothioconazole	6451142	OC(Cc1ccccc1Cl)(Cn1[nH]cnc1=S)C1(Cl)CC1
145	albendazole	2082	CCCSc1ccc2nc(NC(=O)OC)[nH]c2c1
146	benomyl	28780	CCCCNC(=O)n1c(NC(=O)OC)nc2ccccc21
147	carbendazim	25429	COC(=O)Nc1nc2ccccc2[nH]1
148	chlorfenazole	77123	Clc1ccccc1-c1nc2ccccc2[nH]1
149	cypendazole	34280	COC(=O)Nc1nc2ccccc2n1C(=O)NCCCCCC#N
150	debacarb	62208	CCOCCOCCOC(=O)Nc1nc2ccccc2[nH]1
151	dimefluazole	188721	CN(C)S(=O)(=O)n1c(C#N)nc2c(Br)cc(C(F)(F)F)cc21
152	fuberidazole	19756	c1coc(-c2nc3ccccc3[nH]2)c1
153	mecarbinzid	20055210	COC(=O)Nc1nc2ccccc2n1C(=O)NCCSC
154	rabenzazole	38441	Cc1cc(C)n(-c2nc3ccccc3[nH]2)n1
155	thiabendazole	5430	c1ccc2[nH]c(-c3cscn3)nc2c1
156	probenazole	91587	C=CCOC1=NS(=O)(=O)c2ccccc21
157	acibenzolar	10171321	O=C(S)c1cccc2nnsc12
158	bentaluron	4408229	CC(C)NC(=O)Nc1nc2ccccc2s1
159	benthiazole	30692	N#CSCSc1nc2ccccc2s1
160	chlobenthiazone	173964	Cn1c(=O)sc2cccc(Cl)c21
161	triazoxide	93422	[O-][n+]1nc(-n2ccnc2)nc2ccc(Cl)cc21
162	allicin	65036	C=CCS[S+]([O-])CC=C
163	carvacrol	10364	Cc1ccc(C(C)C)cc1O
164	carvone	7439	C=C(C)C1CC=C(C)C(=O)C1
165	osthol	10228	COc1ccc2ccc(=O)oc2c1CC=C(C)C
166	santonin	221071	CC1=C2C3OC(=O)C(C)C3CCC2(C)C=CC1=O
167	berberine	2353	COc1ccc2cc3[n+](cc2c1OC)CCc1cc2c(cc1-3)OCO2
168	sanguinarine	5154	C[n+]1cc2c3c(ccc2c2ccc4cc5c(cc4c21)OCO5)OCO3
169	ofurace	42850	Cc1cccc(C)c1N(C(=O)CCl)C1CCOC1=O
170	vangard	24744	COC(=O)Nc1cccc(OC(=O)Nc2cccc(C)c2)c1
171	iodocarb	62097	CCCCNC(=O)OCC#CI
172	propamocarb	32490	CCCOC(=O)NCCCN(C)C
173	prothiocarb	29697	CCSC(=O)NCCCN(C)C
174	pyribencarb	11577870	COC(=O)NCc1cc(C(C)=NOCc2cccc(C)n2)ccc1Cl
175	diethofencarb	91742	CCOc1ccc(NC(=O)OC(C)C)cc1OCC
176	tolprocarb	57957886	Cc1ccc(C(=O)NCC(NC(=O)OCC(F)(F)F)C(C)C)cc1
177	chlorothalonil	15910	N#Cc1c(Cl)c(Cl)c(Cl)c(C#N)c1Cl
178	cyazofamid	9862076	Cc1ccc(-c2c(Cl)nc(C#N)n2S(=O)(=O)N(C)C)cc1
179	chlozolinate	51574	CCOC(=O)C1(C)OC(=O)N(c2cc(Cl)cc(Cl)c2)C1=O
180	dichlozoline	32253	CC1(C)OC(=O)N(c2cc(Cl)cc(Cl)c2)C1=O
181	dimetachlone	90363	O=C1CCC(=O)N1c1cc(Cl)cc(Cl)c1
182	iprodione	37517	CC(C)NC(=O)N1CC(=O)N(c2cc(Cl)cc(Cl)c2)C1=O
183	isovaledione	155450	CC(C)CC(=O)N1CC(=O)N(c2cc(Cl)cc(Cl)c2)C1=O
184	metomeclan	157855	COCC1CC(=O)N(c2cc(Cl)cc(Cl)c2)C1=O
185	myclozolin	92350	COCC1(C)OC(=O)N(c2cc(Cl)cc(Cl)c2)C1=O
186	procymidone	36242	CC12CC1(C)C(=O)N(c1cc(Cl)cc(Cl)c1)C2=O
187	vinclozolin	39676	C=CC1(C)OC(=O)N(c2cc(Cl)cc(Cl)c2)C1=O
188	fluoroimide	93228	O=C1C(Cl)=C(Cl)C(=O)N1c1ccc(F)cc1

189	captafol	17038	O=C1C2CC=CCC2C(=O)N1SC(Cl)(Cl)C(Cl)Cl
190	captan	8606	O=C1C2CC=CCC2C(=O)N1SC(Cl)(Cl)Cl
191	folpet	8607	O=C1c2ccccc2C(=O)N1SC(Cl)(Cl)Cl
192	thiochlorfenphim	3894809	O=C1c2ccccc2C(=O)N1CSc1ccc(Cl)cc1
193	fluazinam	91731	O=[N+]([O-])c1cc(C(F)(F)F)c(Cl)c([N+](=O)[O-])c1Nc1ncc(C(F)(F)F)cc1Cl
194	dinobuton	13783	CCC(C)c1cc([N+](=O)[O-])cc([N+](=O)[O-])c1OC(=O)OC(C)C
195	dinocton	169442	COC(=O)Oc1c(CCCCCC(C)C)cc([N+](=O)[O-])cc1[N+](=O)[O-]
196	dinopenton	19745588	CCCC(C)c1cc([N+](=O)[O-])cc([N+](=O)[O-])c1OC(=O)OC(C)C
197	dinosulfon	20544356	CCCCCCC(C)c1cc([N+](=O)[O-])cc([N+](=O)[O-])c1OC(=O)SC
198	dinoterbon	14304763	CCOC(=O)Oc1c([N+](=O)[O-])cc([N+](=O)[O-])cc1C(C)(C)C
199	DNOC	10800	Cc1cc([N+](=O)[O-])cc([N+](=O)[O-])c1O
200	binapacryl	10234	CCC(C)c1cc([N+](=O)[O-])cc([N+](=O)[O-])c1OC(=O)C=C(C)C
201	dinocap-4 R1	5290882	CC=CC(=O)Oc1c([N+](=O)[O-])cc(C(C)CCCCCC)cc1[N+](=O)[O-]
202	dinocap-4 R2	18772429	CC=CC(=O)Oc1c([N+](=O)[O-])cc(C(CC)CCCCC)cc1[N+](=O)[O-]
203	dinocap-4 R3	18772430	CC=CC(=O)Oc1c([N+](=O)[O-])cc(C(CCC)CCCC)cc1[N+](=O)[O-]
204	meptyldinocap	5284389	CC=CC(=O)Oc1c(C(C)CCCCCC)cc([N+](=O)[O-])cc1[N+](=O)[O-]
205	azithiram	3032491	CN(C)NC(=S)SSC(=S)NN(C)C
206	dazomet	10788	CN1CSC(=S)N(C)C1
207	disulfiram	3117	CCN(CC)C(=S)SSC(=S)N(CC)CC
208	etem	36603	S=C1SSC2=NCCN12
209	metam	3001858	CNC(=S)S
210	milneb	92200	CC1NC(C)N(CCN2C(=S)SC(C)NC2C)C(=S)S1
211	tecoram	3034167	CN(C)C(=S)SSC(=S)NCCNC(=S)SSC(=S)N(C)C
212	asomate	19167	CN(C)C(=S)S[As](SC(=S)N(C)C)SC(=S)N(C)C
213	carbamorph	35953	CN(C)C(=S)SCN1CCOCC1
214	thiram	5455	CN(C)C(=S)SSC(=S)N(C)C
215	urbacide	17128	CN(C)C(=S)S[As](C)SC(=S)N(C)C
216	isoprothiolane	39681	CC(C)OC(=O)C(C(=O)OC(C)C)=C1SCCS1
217	chloraniformethan	30331	O=CNC(Nc1ccc(Cl)c(Cl)c1)C(Cl)(Cl)Cl
218	trimorphamide	43172	O=CNC(N1CCOCC1)C(Cl)(Cl)Cl
219	allyl isothiocyanate	5971	C=CCN=C=S
220	carbon disulfide	6348	S=C=S
221	chloropicrin	6423	O=[N+]([O-])C(Cl)(Cl)Cl
222	cyanogen	9999	N#CC#N
223	DBCP	7280	ClCC(Br)CBr
224	dimethyl disulfide	12232	CSSC
225	methyl bromide	6323	CBr
226	methyl iodide	6328	CI
227	methyl isothiocyanate	11167	CN=C=S
228	guazatine	3526	NC(N)=NCCCCCCCCNCCCCCCCCN=C(N)N
229	moroxydine	71655	N=C(N=C(N)N)N1CCOCC1
230	benquinox	10320	O=Nc1ccc(NNC(=O)c2ccccc2)cc1

231	chloroinconazide	157010717	CC1NC(C(=O)NN=Cc2ccc(Cl)cc2)Cc2c1[nH]c1ccccc21
232	saijunmao	43990	O=C(NN=C1SCCS1)c1ccccc1
233	fenamidone	10403199	CSC1=NC(C)(c2ccccc2)C(=O)N1Nc1ccccc1
234	phthalide	6885	O=C1OCc2ccccc21
235	dichlobentiazox	17756624	O=S1(=O)N=C(OCc2snc(Cl)c2Cl)c2ccccc21
236	octhilinone	33528	CCCCCCCCn1sccc1=O
237	drazoxolon	21929	Cc1[nH]oc(=O)c1N=Nc1ccccc1Cl
238	hymexazol	24781	Cc1cc(=O)[nH]o1
239	aldimorph	3014032	CCCCCCCCCCCCN1CC(C)OC(C)C1
240	dodemorph	61899	CC1CN(C2CCCCCCCCCCC2)CC(C)O1
241	fenpropimorph	93365	CC(Cc1ccc(C(C)(C)C)cc1)CN1CC(C)OC(C)C1
242	tridemorph	32518	CCCCCCCCCCCCCN1CC(C)OC(C)C1
243	chlorodinitronaphthalenes	16987	O=[N+]([O-])c1cc([N+](=O)[O-])c2ccccc2c1Cl
244	sultropen	13226549	CCCCCS(=O)(=O)c1ccc([N+](=O)[O-])cc1[N+](=O)[O-]
245	thiocyanatodinitrobenzenes 2-4-	15325	N#CSc1ccc([N+](=O)[O-])cc1[N+](=O)[O-]
246	thiocyanatodinitrobenzenes 3-5-	23009548	N#CSc1cc([N+](=O)[O-])cc([N+](=O)[O-])c1
247	trichlorotrinitrobenzenes	17518	O=[N+]([O-])c1c(Cl)c([N+](=O)[O-])c(Cl)c([N+](=O)[O-])c1Cl
248	hexachlorobenzene	8370	Clc1c(Cl)c(Cl)c(Cl)c(Cl)c1Cl
249	hexachlorobutadiene	6901	ClC(Cl)=C(Cl)C(Cl)=C(Cl)Cl
250	EBP	25834	CCOP(=O)(OCC)SCc1ccccc1
251	phosdiphen	92304	CCOP(=O)(Oc1ccc(Cl)cc1Cl)Oc1ccc(Cl)cc1Cl
252	hexylthiofos	6451756	CCOP(=O)(OC1CCCCC1)SCC
253	tolclofos-methyl	91664	COP(=S)(OC)Oc1c(Cl)cc(C)cc1Cl
254	ampropylfos	178368	CCC(N)P(=O)(O)O
255	fosetyl	6328134	CCO[P+](=O)O
256	inezin	30772	CCOP(=O)(SCc1ccccc1)c1ccccc1
257	ditalimfos	21207	CCOP(=S)(OCC)N1C(=O)c2ccccc2C1=O
258	edifenphos	28292	CCOP(=O)(Sc1ccccc1)Sc1ccccc1
259	iprobenfos	33294	CC(C)OP(=O)(OC(C)C)SCc1ccccc1
260	pyrazophos	26033	CCOC(=O)c1cn2nc(OP(=S)(OCC)OCC)cc2nc1C
261	triamiphos	13943	CN(C)P(=O)(N(C)C)n1nc(-c2ccccc2)nc1N
262	metazoxolon	21930	Cc1[nH]oc(=O)c1N=Nc1cccc(Cl)c1
263	famoxadone	213032	CC1(c2ccc(Oc3ccccc3)cc2)OC(=O)N(Nc2ccccc2)C1=O
264	oxadixyl	53735	COCC(=O)N(c1c(C)cccc1C)N1CCOC1=O
265	bithionol	2406	Oc1c(Cl)cc(Cl)cc1Sc1cc(Cl)cc(Cl)c1O
266	o-cresol	335	Cc1ccccc1O
267	m-cresol	342	Cc1cccc(O)c1
268	p-cresol	2879	Cc1ccc(O)cc1
269	dichlorophen	3037	Oc1ccc(Cl)cc1Cc1cc(Cl)ccc1O
270	fenjuntong	76157	CCCC(=O)c1ccccc1O
271	hexachlorophene	3598	Oc1c(Cl)cc(Cl)c(Cl)c1Cc1c(O)c(Cl)cc(Cl)c1Cl
272	pentachlorophenol	992	Oc1c(Cl)c(Cl)c(Cl)c(Cl)c1Cl
273	2-phenylphenol	7017	Oc1ccccc1-c1ccccc1
274	ipflufenoquin	68289416	Cc1nc2c(F)c(F)ccc2cc1Oc1cccc(F)c1C(C)(C)O
275	fenpiclonil	91724	N#Cc1c[nH]cc1-c1cccc(Cl)c1Cl
276	fludioxonil	86398	N#Cc1c[nH]cc1-c1cccc2c1OC(F)(F)O2
277	pencycuron	91692	O=C(Nc1ccccc1)N(Cc1ccc(Cl)cc1)C1CCCC1
278	tecloftalam	53469	O=C(O)c1c(Cl)c(Cl)c(Cl)c(Cl)c1C(=O)Nc1cccc(Cl)c1Cl
279	triforine	33565	O=CNC(N1CCN(C(NC=O)C(Cl)(Cl)Cl)CC1)C(Cl)(Cl)Cl
280	fenpropidin	91694	CC(Cc1ccc(C(C)(C)C)cc1)CN1CCCCC1
281	piperalin	18996	CC1CCCCN1CCCOC(=O)c1ccc(Cl)c(Cl)c1

282	fluoxapiprolin	90169437	CS(=O)(=O)Oc1cccc(Cl)c1C1CC(c2csc(C3CCN(C(=O)Cn4nc(C(F)F)cc4C(F)F)CC3)n2)=NO1
283	oxathiapiprolin	56945145	Cc1cc(C(F)(F)F)nn1CC(=O)N1CCC(c2nc(C3=NOC(c4c(F)cccc4F)C3)cs2)CC1
284	polyoxin B	181352	NC(=O)OCC(O)C(O)C(N)C(=O)NC(C(=O)O)C1OC(n2cc(CO)c(=O)[nH]c2=O)C(O)C1O
285	polyoxins	3084093	NC(C=O)C1OC(n2cc(C(=O)O)c(=O)[nH]c2=O)C(O)C1O
286	polyoxorim	72476	NC(=O)OCC(O)C(O)C(N)C(=O)NC(C(=O)O)C1OC(n2cc(C(=O)O)c(=O)[nH]c2=O)C(O)C1O
287	pyridachlometyl	71615984	Cc1nnc(Cl)c(-c2c(F)cccc2F)c1-c1ccccc1
288	diclomezine	93341	Cc1c(Cl)cc(-c2ccc(=O)[nH]n2)cc1Cl
289	aminopyrifen	91810812	Cc1ccc(C(=O)OCc2ccc(Oc3ccccc3)cc2)c(N)n1
290	buthiobate	39978	CCCCSC(=Nc1cccnc1)SCc1ccc(C(C)(C)C)cc1
291	dipyrithione	3109	[O-][n+]1ccccc1SSc1cccc[n+]1[O-]
292	pyridinitril	14146	N#Cc1c(Cl)nc(Cl)c(C#N)c1-c1ccccc1
293	pyrifenox	55790	CON=C(Cc1cccnc1)c1ccc(Cl)cc1Cl
294	pyrisoxazole	11512926	CN1OC(c2ccc(Cl)cc2)CC1(C)c1cccnc1
295	pyroxychlor	23547	COc1cc(C(Cl)(Cl)Cl)cc(Cl)n1
296	pyroxyfur	129813	Clc1cc(C(Cl)(Cl)Cl)cc(OCc2ccco2)n1
297	parinol	28768	OC(c1ccc(Cl)cc1)(c1ccc(Cl)cc1)c1cccnc1
298	bupirimate	38884	CCCCc1c(C)nc(NCC)nc1OS(=O)(=O)N(C)C
299	dimethirimol	135424353	CCCCc1c(C)nc(N(C)C)[nH]c1=O
300	ethirimol	135424354	CCCCc1c(C)nc(NCC)[nH]c1=O
301	cyprodinil	86367	Cc1cc(C2CC2)nc(Nc2ccccc2)n1
302	mepanipyrim	86296	CC#Cc1cc(C)nc(Nc2ccccc2)n1
303	pyrimethanil	91650	Cc1cc(C)nc(Nc2ccccc2)n1
304	diflumetorim	15390131	CCC(Nc1ncnc(C)c1Cl)c1ccc(OC(F)F)cc1
305	fenarimol	43226	OC(c1ccc(Cl)cc1)(c1cncnc1)c1ccccc1Cl
306	nuarimol	91683	OC(c1ccc(F)cc1)(c1cncnc1)c1ccccc1Cl
307	triarimol	33605	OC(c1ccccc1)(c1cncnc1)c1ccc(Cl)cc1Cl
308	ferimzone	9013425	CC(=NNc1nc(C)cc(C)n1)c1ccccc1C
309	pyroquilon	91665	O=C1CCc2cccc3c2N1CC3
310	fenazaquin	86356	CC(C)(C)c1ccc(CCOc2ncnc3ccccc23)cc1
311	proquinazid	11057771	CCCOc1nc2ccc(I)cc2c(=O)n1CCC
312	ethoxyquin	3293	CCOc1ccc2c(c1)C(C)=CC(C)(C)N2
313	halacrinate	114868	C=CC(=O)Oc1c(Br)cc(Cl)c2cccnc12
314	quinacetol	95877	CC(=O)c1ccc(O)c2ncccc12
315	quinofumelin	23160856	CC1(C)N=C(c2cnc3ccccc3c2)c2ccccc2C1(F)F
316	quinoxyfen	3391107	Fc1ccc(Oc2ccnc3cc(Cl)cc(Cl)c23)cc1
317	tebufloquin	11629002	CC(=O)Oc1c(C)c(C)nc2c(F)cc(C(C)(C)C)cc12
318	chloranil	8371	O=C1C(Cl)=C(Cl)C(=O)C(Cl)=C1Cl
319	dichlone	8342	O=C1C(Cl)=C(Cl)C(=O)c2ccccc21
320	dithianon	18771	N#Cc1sc2c(=O)c3ccccc3c(=O)c=2sc1C#N
321	quinazamid	959141	NC(=O)N=Nc1ccc(O)cc1
322	chinomethionat	17109	Cc1ccc2nc3sc(=O)sc3nc2c1
323	chlorquinox	19014	Clc1c(Cl)c(Cl)c2nccnc2c1Cl
324	thioquinox	7159	S=c1sc2nc3ccccc3nc2s1
325	spiroxamine	86160	CCCN(CC)CC1COC2(CCC(C(C)(C)C)CC2)O1
326	fluoxastrobin	11048796	CON=C(C1=NOCCO1)c1ccccc1Oc1ncnc(Oc2ccccc2Cl)c1F
327	mandestrobin	10935908	CNC(=O)C(OC)c1ccccc1COc1cc(C)ccc1C
328	azoxystrobin	3034285	COC=C(C(=O)OC)c1ccccc1Oc1cc(Oc2ccccc2C#N)ncn1

329	bifujunzhi	102198195	COC(=O)C(=COCF)c1ccccc1COc1nc(Cl)c(Cl)cc1Cl
330	coumoxystrobin	45380432	CCCCc1c(C)c2ccc(OCc3ccccc3C(=COC)C(=O)OC)cc2oc1=O
331	enoxastrobin	59649244	COC=C(C(=O)OC)c1ccccc1CON=C(C)C=Cc1ccc(Cl)cc1
332	flufenoxystrobin	59774441	COC=C(C(=O)OC)c1ccccc1COc1ccc(C(F)(F)F)cc1Cl
333	jiaxiangjunzhi	45381138	COC=C(C(=O)OC)c1ccccc1COc1ccc2c(C)c(C)c(=O)oc2c1
334	picoxystrobin	11285653	COC=C(C(=O)OC)c1ccccc1COc1cccc(C(F)(F)F)n1
335	pyraoxystrobin	45380417	COC=C(C(=O)OC)c1ccccc1COc1cc(-c2ccc(Cl)cc2)nn1C
336	pyraclostrobin	6422843	COC(=O)N(OC)c1ccccc1COc1ccn(-c2ccc(Cl)cc2)n1
337	pyrametostrobin	45381137	COC(=O)N(OC)c1ccccc1COc1c(C)c(-c2ccccc2)nn1C
338	triclopyricarb	57582386	COC(=O)N(OC)c1ccccc1COc1nc(Cl)c(Cl)cc1Cl
339	dimoxystrobin	10936292	CNC(=O)C(=NOC)c1ccccc1COc1cc(C)ccc1C
340	fenaminstrobin	45380430	CNC(=O)C(=NOC)c1ccccc1CON=C(C)C=Cc1c(Cl)cccc1Cl
341	metominostrobin	5483872	CNC(=O)C(=NOC)c1ccccc1Oc1ccccc1
342	orysastrobin	11486133	CNC(=O)C(=NOC)c1ccccc1CON=C(C)C(=NOC)C(C)=NOC
343	kresoxim-methyl	6112114	CON=C(C(=O)OC)c1ccccc1COc1ccccc1C
344	trifloxystrobin	11664966	CON=C(C(=O)OC)c1ccccc1CON=C(C)c1cccc(C(F)(F)F)c1
345	bifemetstrobin	70188842	COC=C(Oc1cc(-c2ccccc2)ccc1C)C(=O)OC
346	metcyclofenstrobin	155699108	COC=C(Oc1cc(C2CCCCC2)ccc1C)C(=O)OC
347	β-sitosterol	222284	CCC(CCC(C)C1CCC2C3CC=C4CC(O)CCC4(C)C3CCC12C)C(C)C
348	amisulbrom	10238657	Cc1c(Br)c2ccc(F)cc2n1S(=O)(=O)c1ncn(S(=O)(=O)N(C)C)n1
349	metyltetraprole	89881183	Cc1cccc(-n2nnn(C)c2=O)c1COc1ccn(-c2ccc(Cl)cc2)n1
350	picarbutrazox	56945144	Cn1nnnc1C(=NOCc1cccc(NC(=O)OC(C)(C)C)n1)c1ccccc1
351	etridiazole	17432	CCOc1nc(C(Cl)(Cl)Cl)ns1
352	flutianil	44151818	COc1ccccc1N1CCSC1=C(C#N)Sc1cc(C(F)(F)F)ccc1F
353	thiadifluor	20056439	CN=C1SC(=NC(F)(F)F)C(=NC(F)(F)F)N1c1ccc(Cl)cc1
354	methasulfocarb	93362	CNC(=O)Sc1ccc(OS(C)(=O)=O)cc1
355	pyributicarb	93486	COc1cccc(N(C)C(=S)Oc2cccc(C(C)(C)C)c2)n1
356	thicyofen	14880662	CC[S+]([O-])c1sc(C#N)c(Cl)c1C#N
357	furophanate	3037184	COC(=O)NC(=S)Nc1ccccc1N=Cc1ccco1
358	thiophanate	3032792	CCOC(=O)NC(=S)Nc1ccccc1NC(=S)NC(=O)OCC
359	thiophanate-methyl	3032791	COC(=O)NC(=S)Nc1ccccc1NC(=S)NC(=O)OC
360	anilazine	7541	Clc1nc(Cl)nc(Nc2ccccc2Cl)n1
361	tricyclazole	39040	Cc1cccc2sc3nncn3c12
362	ametoctradin	15604010	CCCCCCCCc1c(CC)nc2ncnn2c1N
363	mepitriflufenpyr	11158213	CC1CCN(c2c(-c3c(F)cc(F)cc3F)c(Cl)nc3ncnn23)CC1
364	allyl alcohol	7858	C=CCO
365	aminotipyr	172914560	Cc1ccc(C(=O)OCc2ccc(Oc3ncc(C)s3)cc2)c(N)n1
366	bethoxazin	12040175	[O-][S+]1CCON=C1c1cc2ccccc2s1
367	bromothalonil	61948	N#CCCC(Br)(C#N)CBr
368	cetoctaelat	3625611	CCCCCCCCCCCCCCCCOCCOCCOCCOCCOCCOCCOCCOCCO
369	chitosan	71853	COC(=O)NC1C(OC2C(CO)OC(OC3C(CO)OC(O)C(N)C3O)C(N)C2O)OC(CO)C(OC2OC(CO)C(OC3OC(CO)C(OC4OC(CO)C(OC5OC(CO)C(OC6OC(CO)C(OC7OC(CO)C(O)C(O)C7N)C(O)C6N)C(O)C5N)C(O)C4N)C(O)C3N)C(O)C2N)C1O
370	dehydroacetic acid	122903	CC(=O)C1C(=O)C=C(C)OC1=O
371	2,5-dichlorobenzoic acid	5784	O=C(O)c1cc(Cl)ccc1Cl
372	diethyl pyrocarbonate	3051	CCOC(=O)OC(=O)OCC
373	dipymetitrone	5236877	Cn1c(=O)c2sc3c(=O)n(C)c(=O)c3sc2c1=O

374	ethylicin	229052	CCSS(=O)(=O)CC
375	feneptamidoquin	130406050	CC(C)CC(C)(Cc1ccccc1)NC(=O)c1cnc2c(F)cccc2c1
376	fenitropan	93358	CC(=O)OCC(C(OC(C)=O)c1ccccc1)[N+](=O)[O-]
377	flumetylsulforim	90259789	Cc1ccc(S(=O)(=O)n2cc(F)c(=N)n(C)c2=O)cc1
378	formaldehyde	712	C=O
379	furfural	7362	O=Cc1ccco1
380	nitrostyrene	6440121	CC(=Cc1ccc(SC#N)cc1)[N+](=O)[O-]
381	nitrothal-isopropyl	43704	CC(C)OC(=O)c1cc(C(=O)OC(C)C)cc([N+](=O)[O-])c1
382	OCH	19936	O=C1C(Cl)=C(Cl)C(Cl)(Cl)C(Cl)(Cl)C1(Cl)Cl
383	oxyfenthiin	13651848	O=S1(=O)C=C(c2ccccc2)S(=O)(=O)CC1
384	quinaminoprole	165392216	Clc1ccc(-n2ccc(OCCNc3ncnc4ccccc34)n2)cc1
385	dinoseb	6950	CCC(C)c1cc([N+](=O)[O-])cc([N+](=O)[O-])c1O
386	dinoterb	14994	CC(C)(C)c1cc([N+](=O)[O-])cc([N+](=O)[O-])c1O
387	DNOP 2,4-	3032447	CCCCCCC(C)c1cc([N+](=O)[O-])cc([N+](=O)[O-])c1O

Table S1.2 Full names of descriptors d17, corresponding to model RF_BF_17

Code d17	Full name of the QuBiLS-MAS descriptor
d17.1	N1_B_AB_nCi_2_NS4_T_NSRW_a-e_MAS
d17.2	SD_Q_AB_nCi_2_SS2_X_LGP[2-4]_e_MAS
d17.3	S_B_AB_nCi_2_SS1_n_A_NSRW_c-h_MAS
d17.4	I50_F_AB_nCi_2_SS4_T_LGP[1;2;6]_e_MAS
d17.5	HM_B_BB_nCi_2_SS4_H_A_SRW_psa-r_MAS
d17.6	VC_B_AB_nCi_2_NS1_T_LGP[1;2;6]_r-m_MAS
d17.7	RA_B_AB_nCi_2_SS2_A_SRW_c-h_MAS
d17.8	MX_B_AB_nCi_2_SS1_C_LGP[1]_c-m_MAS
d17.9	N3_B_AB_nCi_2_NS1_H_T_KA_p-h_MAS
d17.10	N3_F_AB_nCi_2_NS1_A_NSRW_c_MAS
d17.11	P2_Q_BB_Ci(2.0;-2.0)_2_NS5_H_A_LGP[2-4]_h_MAS
d17.12	N3_B_AB_nCi_2_MP1_A_NSRW_e-c_MAS
d17.13	GM_B_AB_nCi_2_SS7_A_LGP[1;2;6]_psa-s_MAS
d17.14	MN_F_AB_nCi_2_SS1_H_n_X_NSRW_c_MAS
d17.15	N1_B_AB_nCi_2_MP8_P_LGP[4-6]_psa-v_MAS
d17.16	MN_F_AB_nCi_2_SS1_T_LGP[1;2;6]_c_MAS
d17.17	S_B_AB_nCi_2_NS8_X_LGP[2-4]_e-c_MAS

Table S1.3 Full names of descriptors d15, corresponding to models RF_BF_15 and RF_BF_14

Code d15	Full name of the QuBiLS-MAS descriptor
d15.1	S_B_AB_nCi_2_NS2_T_NSRW_psa-c_MAS
d15.2	S_Q_AB_nCi_2_MP5_A_LGP[1;2;6]_a_MAS
d15.3	MN_B_AB_nCi_2_SS3_H_n_X_LGP[2-4]_a-h_MAS
d15.4	S_B_AB_nCi_2_SS7_T_KA_e-s_MAS
d15.5	N1_B_AB_nCi_2_MP7_n_T_KA_h-s_MAS
d15.6	N3_F_AB_nCi_2_NS1_A_NSRW_c_MAS
d15.7	MX_B_AB_nCi_2_NS7_C_LGP[1]_c-h_MAS
d15.8	P2_B_AB_Ci(2.0;-2.0)_2_SS3_H_A_LGP[1]_c-m_MAS
d15.9	MX_B_AB_nCi_2_SS4_X_SRW_psa-h_MAS
d15.10	MN_B_AB_nCi_2_NS6_H_n_X_LGP[2-4]_a-psa_MAS
d15.11	HM_B_AB_nCi_2_SS2_X_KA_h-s_MAS
d15.12	MN_F_AB_nCi_2_SS1_H_n_X_NSRW_c_MAS
d15.13	K_B_AB_nCi_2_NS7_X_LGP[2-4]_a-c_MAS
d15.14	N3_Q_AB_nCi_2_SS1_X_LGP[1]_a_MAS
d15.15	N3_B_AB_nCi_2_SS1_H_T_KA_c-m_MAS
