# Supplementary material for: FLiP-Z by Zimeck: A Python-Based Machine-Learning Tool for Predicting Fungicide-Likeness of Organic Molecules
Source: Int J Mol Sci. 2026 Aug 24;27(17):7562. doi: 10.3390/ijms27177562 (PMC13566103; doi:10.3390/ijms27177562)
Supplement: Supplementary file 1 [file ijms-27-07562-s001.zip › SupplementaryInformation_S6.pdf]

Supplementary Information S6:

Multi-factor PerMANOVA calculations for external validation data

1. Rationale for Multivariate Statistical Validation

The strategic importance of performing a robust external validation lies in its ability to evaluate the true predictive power of QSAR models when applied to independent test datasets. Traditional univariate tests often fail to capture the holistic performance of these models, as metrics such as Accuracy (ACC), Sensitivity (SENS), Specificity (SPEC), Matthews' Correlation Coefficient (MCC), and the Area Under the Receiver Operating Characteristic Curve (ROC-AUC) are inherently correlated. Because these five metrics represent different dimensions of a singular outcome and are derived from the same underlying contingency table data, any change in model parameters simultaneously impacts the entire performance profile. To address this, a multivariate approach using Permutational Analysis of Variance (PerMANOVA) was selected. Unlike traditional parametric tests, PerMANOVA's permutation-based design does not require parametric error terms, making it uniquely suited for deterministic computational data where random experimental error is absent. This methodology ensures that the statistical significance of observed differences is measured with high academic rigor, providing a comprehensive assessment of the experimental factors.

2. Experimental Design and Input Data

To rigorously assess the factors influencing model performance, a 3-factor experimental design was implemented. This design allows for the simultaneous evaluation of how the choice of molecular descriptors, the origin of the chemical data, and the balance of the dataset affect the overall predictive success of the optimized models.The following table reconstructs the raw performance metrics for the twelve unique experimental combinations identified in the study:

Table S6.1 External Validation Data: Performance Metrics

Descriptor Type	Data Origin	Dataset Balance	ACC	SENS	SPEC	MCC	ROC-AUC
QuBiLS-MAS	Agrochemicals	Balanced	0.806	0.778	0.828	0.605	0.861
QuBiLS-MAS	Agrochemicals	Moderately unbalanced	0.826	0.489	0.951	0.522	0.881
QuBiLS-MAS	Agrochemicals	Highly unbalanced	0.877	0.352	0.979	0.463	0.882
QuBiLS-MAS	Drugs	Balanced	0.937	0.943	0.933	0.872	0.981
QuBiLS-MAS	Drugs	Moderately unbalanced	0.901	0.750	0.955	0.737	0.965
QuBiLS-MAS	Drugs	Highly unbalanced	0.937	0.700	0.980	0.742	0.945
PhysChem	Agrochemicals	Balanced	0.663	0.527	0.769	0.307	0.711
PhysChem	Agrochemicals	Moderately unbalanced	0.702	0.209	0.886	0.121	0.699
PhysChem	Agrochemicals	Highly unbalanced	0.820	0.121	0.954	0.117	0.691
PhysChem	Drugs	Balanced	0.724	0.692	0.748	0.439	0.788
PhysChem	Drugs	Moderately unbalanced	0.725	0.527	0.798	0.320	0.773
PhysChem	Drugs	Highly unbalanced	0.783	0.396	0.855	0.234	0.770

ACC: Accuracy; SENS: Sensitivity; SPEC: Specificity; MCC: Matthews' Correlation Coefficient; ROC-AUC: Area Under the Receiver Operating Characteristic Curve

The variables are categorized as follows:

- Descriptor Type: Compares the performance of QuBiLs-MAS descriptors against traditional PhysChem descriptors.

- Data Origin: Distinguishes between models trained on Agrochemical-based data versus Drug-based data.
- Dataset Balance: Evaluates the impact of class distribution across three levels: Balanced, Moderately Unbalanced, and Highly Unbalanced.

These structured inputs provide the basis for the subsequent computational analysis performed in a multi-language environment.

3. Computational Implementation and Code Scheme

The analysis required the integration of R's specialized statistical libraries within a Python-based workflow to ensure the absolute highest level of academic rigor. To achieve a true R-style marginal testing (equivalent to Type III Sum of Squares for distances), where all factors are assessed simultaneously in a single linear-like model formula, we utilized the rpy2 bridge to access the vegan::adonis2 function.

The following code demonstrates the setup of the design matrix, the distance method selection, and the execution of the PerMANOVA:

```
import numpy as np
import pandas as pd
from rpy2.robjects.packages import importr
import rpy2.robjects as robjects
from rpy2.robjects import pandas2ri

# 1. Define experimental design and performance data
descriptors = ['qubilsmas', 'physchem']
origins = ['agrochemicals', 'drugs']
treatments = ['balanced', 'moderately_unbalanced', 'highly_unbalanced']

design_matrix = [
  {"Descriptor_Type": d, "Data_Origin": o, "Dataset_Balance": t}
  for d in descriptors for o in origins for t in treatments
]
df_design = pd.DataFrame(design_matrix)

# Input performance metrics derived from Weka evaluations
acc=[0.806,0.826,0.877,0.937,0.901,0.937,0.663,0.702,0.820,0.724,0.725,0.783]
sens=[0.778,0.489,0.352,0.943,0.750,0.700,0.527,0.209,0.121,0.692,0.527,0.396]
spec=[0.828,0.951,0.979,0.933,0.955,0.980,0.769,0.886,0.954,0.748,0.798,0.855]
mcc=[0.605,0.522,0.463,0.872,0.737,0.742,0.307,0.121,0.117,0.439,0.320,0.234]
roc=[0.861,0.881,0.882,0.981,0.965,0.945,0.711,0.699,0.691,0.788,0.773,0.770]

df_performance = pd.DataFrame({
  'Accuracy': acc, 'Sensitivity': sens, 'Specificity': spec, 'MCC': mcc,
'ROC_AUC': roc
})

# 2. Execution of Multi-factor PerMANOVA via R-vegan
vegan = importr('vegan')
pandas2ri.activate()

r_design = robjects.conversion.py2rpy(df_design)
r_perf = robjects.conversion.py2rpy(df_performance)

robjects.globalenv['design'] = r_design
```

```
robjects.globalenv['perf'] = r_perf

# 'euclidean' method is selected as metrics (ACC, MCC, etc.) are on a standardized 0-1 scale.
# 'by=margin' ensures Type III Sum of Squares equivalent marginal testing.

r_code = "adonis2(perf ~ Dataset_Balance + Data_Origin + Descriptor_Type, data=design, method='euclidean', by='margin')"

result = robjects.r(r_code)

print(result)

# Cleanup step to ensure environment remains clean
pandas2ri.deactivate()
```

The execution of this script generates the statistical output required to quantify the specific contribution of each experimental driver.

4. Multi-factor PerMANOVA Results

The PerMANOVA output quantifies the specific contribution of each experimental factor to the total multivariate variance observed in the performance metrics. By examining the R^2 values, we can determine the explanatory power of each component of the experimental design.

Table S6.2 PerMANOVA output

	Df	SumOfSqs	R^2	F	Pr(>F)	
Dataset_Balance	2	0.35336	0.21736	22.674	0.001	***
Data_Origin	1	0.34694	0.21341	44.524	0.001	***
Descriptor_Type	1	0.87084	0.53567	111.758	0.001	***
Residual	7	0.05455	0.03355			
Total	11	1.62568	1.00000			

Signif. codes: 0 '***' 0.001 '**' 0.01 '*' 0.05 '.' 0.1 ' ' 1

The results indicate that all three factors are highly significant ($p = 0.001$), contributing to a comprehensive model that explains the vast majority of the observed variance in model performance.

5. Interpretation of Statistical Drivers and Conclusions

The multi-factor PerMANOVA successfully captured 96.6% of the total multivariate variance, leaving a negligible residual, which underscores the high degree of control within the experimental design.

- Descriptor Type: Accounting for 53.6% of the total variance ($R^2 = 0.53567$), the choice of descriptor is the single most critical factor. Switching from PhysChem to QuBiLs-MAS descriptors fundamentally shifts the performance profile, demonstrating that the structural representation of the molecule is a more potent determinant of model quality than either the balance of the training set or the origin of the data.
- Dataset Balance: This factor contributed 21.7% to the variance ($R^2 = 0.21736$). The highly significant p-value confirms that class imbalance significantly impacts overall performance; as the

imbalance ratio grows, the system's performance dimensions are altered, particularly impacting sensitivity and MCC while specificity increases.

- Data Origin: Explaining 21.3% of the variance (R^2 = 0.21341), the origin of the negative-class instances plays a major role. This confirms that agrochemical-associated models provide a "stricter" context for evaluation than drug-associated models, a result of the greater similarity between the agrochemical negative class and the fungicides being targeted.
- Residual Variance: The residual variance is exceptionally low at 3.4% (R^2 = 0.03355). While "real-world" laboratory experiments typically yield residual errors in the 20–40% range due to human error and environmental noise, this 3.4% score is considered perfect for deterministic computational calculations. It proves that the three chosen factors capture nearly everything happening within the performance metrics.

In conclusion, the multi-factor PerMANOVA provides a robust statistical foundation for the external validation results. It confirms that while all experimental factors are significant, the superiority of QuBiLs-MAS descriptors remains the primary driver of high-performance QSAR outcomes in this study.
